# Maintenance of Cotton Leaf Curl Multan Betasatellite by *Tomato Leaf Curl New Delhi Virus—*Analysis by Mutation

**DOI:** 10.3389/fpls.2017.02208

**Published:** 2017-12-22

**Authors:** Zafar Iqbal, Muhammad Shafiq, Irfan Ali, Shahid Mansoor, Rob W. Briddon

**Affiliations:** ^1^Agricultural Biotechnology Division, National Institute for Biotechnology and Genetic Engineering, Faisalabad, Pakistan; ^2^Pakistan Institute of Engineering and Applied Sciences, Nilore, Pakistan

**Keywords:** ToLCNDV, CLCuMuB, begomovirus, mutation, movement

## Abstract

Viruses of the genus *Begomovirus* (family *Geminiviridae*) are economically important phytopathogens that are transmitted plant-to-plant by the whitefly *Bemisia tabaci*. Most Old World (OW) begomoviruses are monopartite and many of these interact with symptoms and host range determining betasatellites. *Tomato leaf curl New Delhi virus* (ToLCNDV) is one of only a few OW begomoviruses with a bipartite genome (components known as DNA A and DNA B). Four genes [AV2, coat protein (CP), transcriptional-activator protein (TrAP), and AC4] of ToLCNDV were mutated and the effects of the mutations on infectivity, symptoms and the ability to maintain Cotton leaf curl Multan betasatellite (CLCuMuB) were investigated. Infectivity and virus/betasatellite DNA titer were assessed by Southern blot hybridization, PCR, and quantitative PCR. The results showed TrAP of ToLCNDV to be essential for maintenance of CLCuMuB and AV2 to be important only in the presence of the DNA B. AC4 was found to be important for the maintenance of CLCuMuB in the presence of, but indispensable in the absence of, the DNA B. Rather than being required for maintenance, the CP was shown to possibly interfere with maintenance of the betasatellite. The findings show that the interaction between a bipartite begomovirus and a betasatellite is more complex than just trans-replication. Clearly, multiple levels of interactions are present and such associations can cause additional significant losses to crops although the interaction may not be stable.

## Introduction

Viruses belonging to family *Geminiviridae* have circular, single-stranded (ss)DNA genomes of ~2.8–5.6 kb encapsidated in twinned quasi-icosahedral particles. The family *Geminiviridae* has been expanded recently to include nine genera (*Begomovirus, Capulavirus, Curtovirus, Grablovirus, Mastrevirus, Topocuvirus, Becurtovirus, Eragrovirus*, and *Turncurtovirus*) (Brown et al., 2012; Adams et al., 2013; Zerbini et al., 2017) and the genus *Begomovirus* comprises the most destructive viruses that are transmitted by the whitefly *Bemisia tabaci*. Typically the genomes of begomoviruses native to the New World (NW) consist of two components, designated as DNA A and DNA B, and both these components are required for virus infectivity. However, recently a monopartite begomovirus, having a single component genome, homologous to the DNA A component of the bipartite viruses, has been identified in the NW (Melgarejo et al., 2013; Sánchez-Campos et al., 2013). In contrast, monopartite begomoviruses are more prevalent in the Old World (OW), with only a few bipartite begomoviruses having been characterized. Additionally, the majority of monopartite begomoviruses have been shown to associate with a class of symptom-modulating satellites known as betasatellites.

In common with all geminiviruses the genes encoded by the genomes (or genomic components) of begomoviruses are expressed from both DNA strands and diverge from a non-coding intergenic region (IR). The DNA A of bipartite and genomes of monopartite begomoviruses encode on the virion-sense strand the (A)V2 protein [involved in movement of the virus in plants (Rojas et al., 2001)] and the coat protein (CP; involved in *in planta* movement, and insect transmission between plants; Rojas et al., 2005). The complementary-sense strand encodes the replication-associated protein [Rep; the only virus-encoded protein required for replication of the virus genome, a rolling circle replication (RCR) initiator protein, as well as a suppressor of transcriptional gene silencing (TGS) Hanley-Bowdoin et al., 2004; Rodríguez-Negrete et al., 2013], the transcriptional-activator protein [TrAP; which up-regulates the late, virion-sense genes (Sunter and Bisaro, 1997), modulates host gene expression (Hao et al., 2003; Wang et al., 2003; Buchmann et al., 2009; Baliji et al., 2010; Castillo-González et al., 2015; Kumar et al., 2015), overcomes virus induced hypersensitive cell death (Hussain et al., 2007; Mubin et al., 2010), and is a suppressor of host defense mediated by PTGS (Yang et al., 2007)], the replication-enhancer protein (REn; helps in establishing a favorable environment for virus replication; Settlage et al., 2005) and the C4 protein (a suppressor of PTGS and pathogenicity determinant; Vanitharani et al., 2004; Gopal et al., 2007; Saeed et al., 2008). The proteins encoded on the DNA B component [the virion-sense encoded nuclear shuttle protein (NSP) and the complementary-sense encoded movement protein (MP)] are involved in cell-to-cell and long-distance movement of the virus (Noueiry et al., 1994).

The intergenic region (IR) comprises promoter elements as well as the origin of virion-strand DNA replication, consisting of a hairpin structure with the conserved, between most geminiviruses, nonanucleotide sequence (TAATATT/AC) and, adjacent to the TATA box of the Rep promoter, repeated sequences known as iterons (Hanley-Bowdoin et al., 1999). Iterons are Rep binding sequences to which Rep binds prior to introducing a nick within the nonanucletide sequence to initiate RCR of the virion-strand (Gladfelter et al., 1997). The iterons of viruses differ such that the Rep of one species will usually not initiate replication of the genome of an unrelated virus (Argüello-Astorga et al., 1994). A sequence, known as the common region, is shared between the two genomic components of bipartite begomoviruses and usually resides within the IR (Stanley and Gay, 1983). This ensures that each component has an origin of replication which will be recognized by the Rep encoded on the DNA A component, maintaining the integrity of the split genome.

Betasatellites are small (~1,350 nt) ssDNA satellites that are most commonly associated with monopartite begomoviruses. Recently a betasatellite has also been identified in association with the leafhopper transmitted *Wheat dwarf India virus* of the genus *Mastrevirus* (family *Geminiviridae*) in a monocotyledonous host (Kumar et al., 2014). Betasatellites have so far only been identified in the OW (Briddon and Mansoor, 2008) although a group of satellites derived from betasatellites, collectively known as delatsatellites, have been shown also to be present in the NW (Lozano et al., 2016). Although only first identified in 1999 (Saunders et al., 2000), the full-length sequences of greater than 1,000 betasatellites have to date been deposited in the databases, indicating the importance of these molecules to agriculture in the warmer parts of the World. Betasatellites require a helper virus for replication and movement in, as well as transmission between, host plants (Briddon et al., 2003). In many instances betasatellites have been found to enhance virus DNA levels in plants as well extending the host-range of the helper virus (Saunders et al., 2000; Briddon et al., 2001). For example, the monopartite begomovirus *Cotton leaf curl Multan virus* (CLCuMuV), one of a number of begomoviruses causing cotton leaf curl disease (CLCuD) in Southern Asia, is poorly infectious to cotton (*Gossypium hirsutum*) and induces non-symptomatic infections (Briddon et al., 2000). In the presence of the betasatellite Cotton leaf curl Multan betasatellite (CLCuMuB), CLCuMuV is highly infectious to cotton and induces typical CLCuD symptoms (Briddon et al., 2001).

The single protein, βC1, encoded by betasatellites is a suppressor of both PTGS and TGS (Zhou, 2013), extends virus host range (Amin et al., 2010), localize and co-localize at endoplasmic reticulum and cell periphery and thus presumably is involved in virus movement in host plants (Saeed et al., 2007), increases DNA levels of the helper begomovirus (Briddon et al., 2001; Iqbal et al., 2012) modulates the levels of microRNAs involved in host developmental processes (Amin et al., 2011b), is a dominant pathogenicity/symptom determinant (Saeed et al., 2005; Qazi et al., 2007), not only interacts with the virus-encoded CP but also with many host-encoded factors (Cheng et al., 2011), binds in a sequence independent manner to DNA and RNA (Cui et al., 2005), and suppresses host jasmonic acid production (Zhang et al., 2012). The sequences of betasatellites encode a predicted hairpin structure with, in most cases, a geminivirus-like nonanucleotide sequence. Betasatellites are true satellites and thus depend entirely on helper virus-encoded Rep to initiate RCR. In most cases betasatellites lack the iterons of their helper viruses. Although the precise interactions between the virus-encoded Rep and the betasatellite DNA required to initiate satellite RCR is unclear (Saunders et al., 2008), it has been suggested that betasatellites have sequences which mimic iterons to allow Rep binding (Nawaz-ul-Rehman et al., 2009).

*Tomato leaf curl New Delhi virus* (ToLCNDV) is a typical OW bipartite begomovirus endemic to South and Southeast Asia. Recently it has also been identified in southern Europe and North Africa (Juarez et al., 2014; Mnari-Hattab et al., 2015; Panno et al., 2016). Although ToLCNDV is believed to be a significant pathogen of tomato in India and Pakistan, the virus has a wide host range (Hussain et al., 2004; Tahir and Haider, 2005; Haider et al., 2006; Ito et al., 2008; Akhter et al., 2009; Mizutani et al., 2011; Nagendran et al., 2016; Srivastava et al., 2016). With increasing frequency ToLCNDV, and other bipartite begomoviruses, are being identified in association of betasatellites most probably due to co-infections between bipartite and betasatellite requiring monopartite begomoviruses (Akhter et al., 2009; Ilyas et al., 2010; Jyothsna et al., 2013; Anwar, 2017). Recently cotton in Pakistan exhibiting CLCuD symptoms has been shown to be extensively infected by ToLCNDV (Zaidi et al., 2016). The significance and effects of the association of betasatellites with bipartite begomoviruses is unclear. The only study so far to address this issue concluded that there is an “antagonism” between the DNA B and betasatellite components, suggesting that the interaction is not stable (Jyothsna et al., 2013).

A previous study has investigated the requirements for maintenance of a betasatellite by mutagenesis of the genes of a monopartite begomovirus—specifically the CLCuD-associated *Cotton leaf curl Kokhran virus* and CLCuMuB (Iqbal et al., 2012). In light of the occasional association of betasatellites with bipartite begomoviruses it seemed timely to investigate the requirements for maintenance of a betasatellite by a bipartite begomovirus. The study described here has investigated the effects of the mutagenesis of selected genes on symptoms and infectivity of the bipartite ToLCNDV and also assessed the effects of the mutations on the maintenance of the betasatellite, CLCuMuB.

## Materials and methods

### Mutagenesis by PCR

A clone of the DNA A component of ToLCNDV (acc. no. U15015) was used to produce specific gene mutants using mutagenic, abutting oligonucleotide primers in PCR (Table 1; Padidam et al., 1995). To mutate the AV2 and TrAP genes, areas of the genes not overlapping the CP, Rep, and REn genes were mutated. Additionally, an extra nucleotide was introduced into AV2 and CP primers to introduce a frame-shift. Mutation of the AC4 gene was accomplished by introducing a stop codon that did not alter the amino acid sequence of the Rep gene with which it overlaps. Mutated full-length virus clones in the plasmid vector pTZ57R/T (InsTAclone PCR Cloning kit, Thermo Fischer Scientific) were completely sequenced to ensure the absence of secondary (unwanted) mutations.

**Table 1:** Sequences of oligonucleotide primers used in mutagenesis, amplification, detection, and quantification of virus components.

**Primer**	**Sequence (5′-3′)^$^**	**Comments**
ToLNDmV2F	CTCGAGACACAGTCGGCTAgGATC	Mutate AV2
ToLNDmV2R	CTCGAGAATAGTTCTTTTATATCTC	
ToLNDmCPF	ATCGATTAGGGTAGCGATTCTaGTGTG	Mutate CP
ToLNDmCPR	ATCGATCGCGATGTGTGAGTCCAGTTC	
mC2TOLNDF	CTCGAGATGTACTACGAACAACCAC	Mutate TrAP
mC2TOLNDR	CTCGAGCAACTGACATGATCACG	
mC4ToLNF	CTCGAGTTGGATCAGAACATGGATATGC	Mutate AC4
mC4ToLNR	CTCGAGGGGAAATTCCAGTGCAAAAATAAC	
ToLNV2pvx/35sF	GGTCGACAAACATGTGGGATCC	Amplify AV2
ToLNV2pvx/35sR	CCCGGGCTTCTATACATTCTGTAC	
PadCPPVXF	GCAAATCGATATGGCGAAGCGACCAG	Amplify CP
PadCPPVXR	GGTCGACTATTAATTTGTGGCCGAATC	
ToLNC2pvx/35F	CAAGTCGACATGCAGTCTTCATC	Amplify TrAP
ToLNC2pvx/35R	ATCCCGGGACTTAAGGACCTGG	
ToLNC4pvx/35F	CGTCGACAAGATGGGTCTCCGC	Amplify AC4
ToLNC4pvx/35R	CCCGGGTCTAGAACGTCTCCATC	
BetaC1F	ATAAATCGATATGACAACGAGCGGAACAAA	Amplify βC1
BetaC1R	TGTTCCCGGGTTAAACGGTGAACTTTTTATT	
BegomoqPCRF1	ATGTGGGATCCACTGTTAAATGAGTTCCC	TA qPCR
BegomoqPCRR1	GATTATATCTGCTGGTCGCTTCGACATAA	
BetaqPCRF2	CAAGTATATCAAGTCTGTGAACTATATCTT	betasatellite qPCR
BetaqPCRR2	GATACTATCCACAAAGTCACCATCGCTAAT	
Beta01	GGTACCACTACGCTACGCAGCAGCC	Amplify betasatellite
Beta02	GGTACCTACCCTCCCAGGGGTACA	
Tr03F	TCTGCCCTATCAACTTTCGATGGTA	18S rDNA qPCR
Tr04R	AATTTGCGCGCCTGCTGCCTTCCTT	
BMPqPCRF	GCCCATGATTCGTTCGGAC	DNA B qPCR
BMPqPCRR	GAATTCCGACCACCAAAGAT	

$ *The underlined nucleotides were changed to introduced a premature stop codon and lower case nucleotides were added to introduce a frame-shift*.

### Production of constructs for infectivity

A partial direct repeat construct of the DNA A component of ToLCNDV harboring a mutation of the AV2 gene (TA^ΔAV2^) was produced by cloning a ~900 bp *Xho*I and *Pst*I fragment in pGreen0029 (Hellens et al., 2000). Then the full-length *Xho*I insert of the pTZ57R/T clone was ligated into the unique *Xho*I restriction site of the pGreen0029 clone containing the partial clone. The full-length PCR amplified product bearing the mutation of the CP (TA^ΔCP^) was digested at the introduced *Mlu*I restriction site, circularized by ligation, digested with *Xba*I and cloned in the binary vector pGreen0029. The resultant clone was digested with *Mlu*I, and the full-length *Mlu*I insert of the pTZ57R/T clone inserted to yield a full dimer. A partial head-to-tail dimer of ToLCNDV DNA A harboring a mutation of the TrAP gene (TA^ΔAC2^) was produced by digestion with *Xho*I and *Pst*I, releasing a fragment of ~1,400 bp which was cloned in pGreen0029. This partial clone was digested with *Xho*I and then the full-length clone, digested with *Xho*I, was inserted. A construct for AC4 gene mutation of ToLCNDV DNA A (TA^ΔAC4^) was similarly produced using a ~550 bp *Xho*I and *BamH*I fragment. The production of a construct for the infectivity of CLCuMuB (acc. no. AJ298903; Briddon et al., 2001), has been described previously (Saeed et al., 2005).

### *Agrobacterium*-mediated inoculation

Constructs for infectivity in binary vectors produced in this study were electroporated into *Agrobacterium tumefaciens* (strain GV3101 or LBA 4404. *Agrobacterium-*mediated inoculation into *N. benthamiana* plants was performed as described previously (Hussain et al., 2004, 2005). Plants were maintained as described previously (Iqbal et al., 2012).

### PCR-mediated diagnostics and southern blot hybridization

Total genomic DNA was extracted from inoculated and control plants by the CTAB method (Doyle and Doyle, 1990). TA was detected by PCR using CP or AV2 primers and CLCuMuB using primers beta01/beta02 (Table 1). For Southern blotting, 10 μg of DNA extracted from plants was electrophoresed in 1.5% agarose gels and then transferred to nylon membranes (Hybond XL, Amersham) by capillary transfer (Sambrook et al., 1989). TA was detected using PCR-amplified, digoxigenin (DIG)-labeled (Roche, Germany) probes to the IR (primers ToLNC4pvx/35R and ToLNV2pvx/35R) and/or TrAP gene (primers ToLNC2pvx/35F and ToLNC2pvx/35R), whereas CLCuMuB (Cβ) was detected using a βC1 gene probe (primers BetaC1F/BetaC1R). ToLCNDV DNA B (TB) was detected on Southern blots using a PCR amplified radioactively labeled (MBI Fermentas, DecaLabel ™ DNA Labeling Kit) MP gene probe as described previously (Dalakouras et al., 2009). Hybridization was conducted at 50°C for 16 h and signals were detected on X-ray film (Super RX, Fuji film) after treating with CDP-Star (Roche, Germany), while a phosphoimager (PharosFX™ Systems Life Science Research Bio-Rad) was used to detect radioactive signals on blots.

### Quantification of viral components by quantitative real-time PCR

The quantity and quality of isolated genomic DNA was assessed using a NanoDrop ND-1000 spectrophotometer (Thermo Fisher Scientific) and the concentration was adjusted to 10 ng/μL. Reactions were conducted in an iCycler Thermal Cycler with iQ5 Multicolor Real-Time PCR Detection System (Bio Rad). The thermal cycler conditions were 94°C for 10 min followed by 40 cycles of 94°C for 30 s (s), 57°C for 30 s, and 72°C for 30 s. Reactions were performed in triplicate on 96 well-plates with a negative control (molecular grade water) and a positive control (ToLCNDV DNA A, DNA B, and CLCuMuB as standards). Amplification of the 18S ribosomal RNA gene was included to normalize for differences in DNA concentrations between samples. At the end of every run, in order to assess the specificity of the amplified product, a melting curve was performed from 57 to 95°C with an increment of 0.5°C every 10 s. Real time PCR assay reactions consisted of 2.5 μL (25 ng) of template DNA, 12.5 μL of SYBR Green Supermix (Thermo Fischer Scientific), 0.25 μL (0.01 pM) of each primer (Table 1) and 9.5 μL of sterile distilled water. Ten-fold serial dilutions of plasmids bearing TA, TB, and Cβ clones were used to obtain standard curves. Serial dilutions of plasmids were spiked with the equal amount of healthy *N. benthamiana* genomic DNA and then analyzed in triplicate. Mean Ct values were used to calculate DNA titers using the standard curves.

### Statistical analysis

Chi-square procedure was used to test the equality of the proportions of infected (out of total inoculated) plants. Significance was observed at 5% level and binomial nomenclature (A and B) was used to show significance between different mutants (Supplementary Table 2). These statistical analyses were carried out by using “R-statistical and computing tool” (R Development Core Team, 2016).

## Results

### Effects on infectivity, symptoms, and the ability to maintain CLCuMuB of mutation of the AV2 gene of ToLCNDV

**Figure 1:**
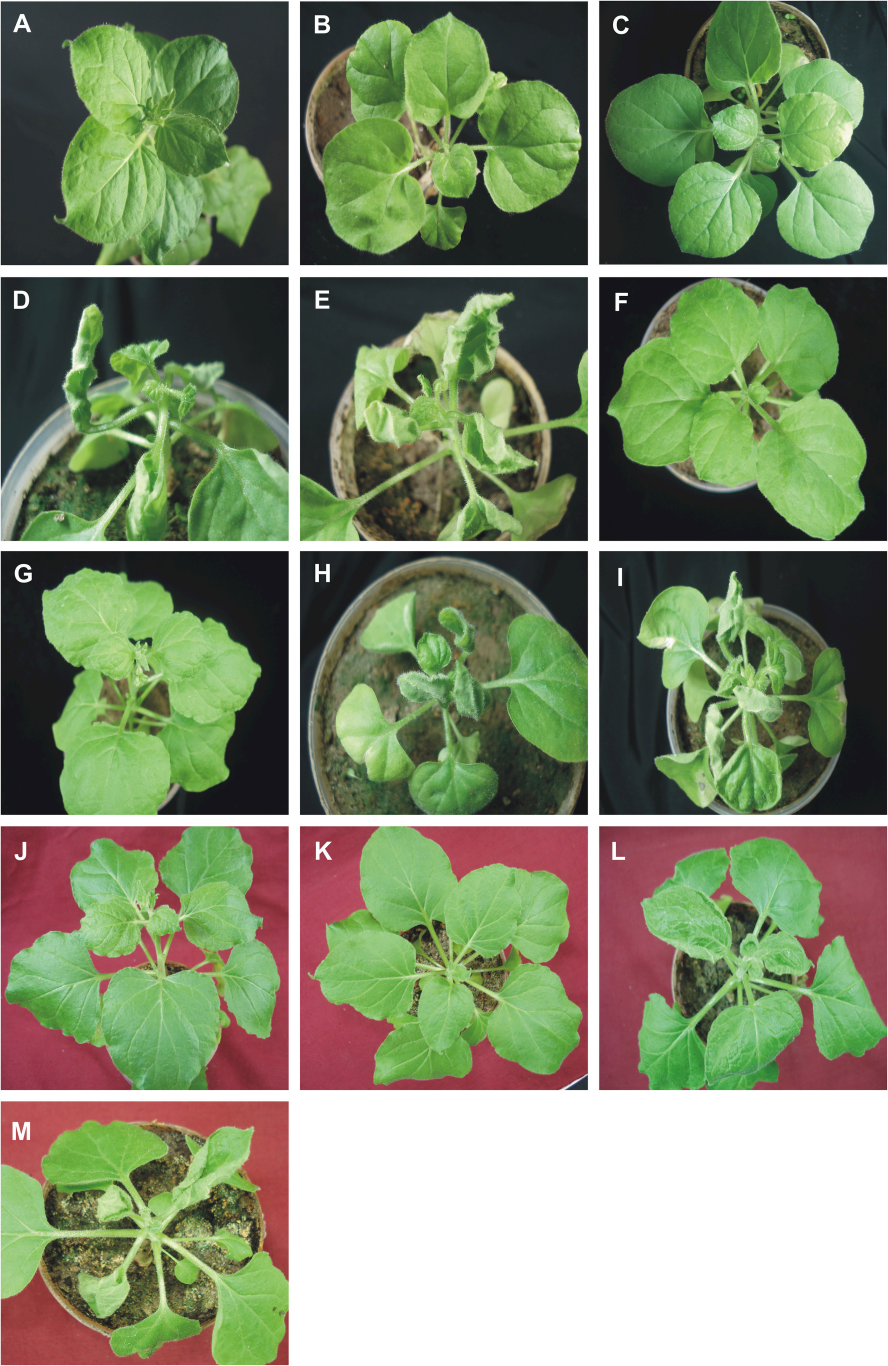
Symptoms in *N. benthamiana* plants following inoculation with ToLCNDV, bearing mutations of the AV2 and CP genes, in the presence and absence of CLCuMuB. The *N. benthamiana* plants shown were either not inoculated (healthy; **A**) or inoculated with TA **(B)**, TA and Cβ **(C)**, TA and TB **(D)**, TA, TB, and Cβ **(E)**, TA^ΔV2^ **(F)**, TA^ΔV2^ and Cβ **(G)**, TA^ΔV2^ and TB **(H)**, TA^ΔV2^, TB, and Cβ **(I)**, TA^ΔCP^ **(J)**, TA^ΔCP^ and Cβ **(K)**, TA^ΔCP^ and TB **(L)**, TA^ΔCP^, TB, and CLCuMuB **(M)**. Photographs were taken at 25 dpi.

**Figure 2:**
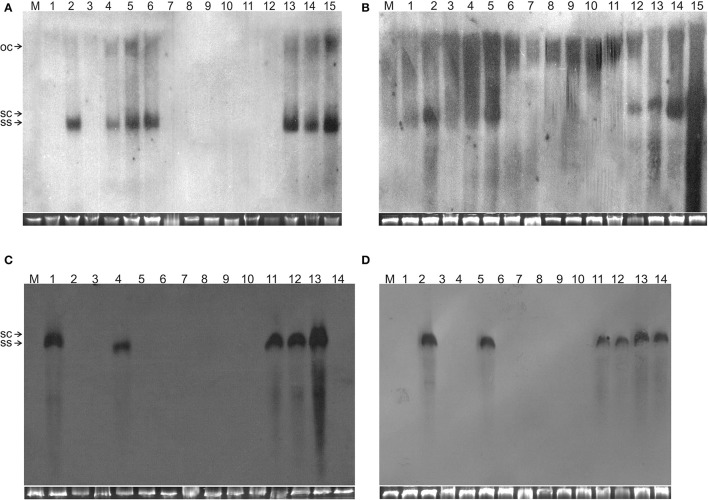
Detection of TA in *N. benthamiana* plants by Southern blot hybridization. The DNA samples resolved on the gels were isolated from leaves of a mock-inoculated plant (lane M) or plants inoculated with **(A)** TA (lane 1), TA and TB (lane 2), TA^ΔAV2^ (lane 3), TA^ΔAV2^ and TB (lanes 4–5), TA, TB and Cβ (lane 6), TA^ΔAV2^ and Cβ (lanes 7–9), TA and Cβ (lanes 10–12) TA^ΔAV2^, TB and Cβ (13–15), **(B)** TA (1), TA and TB (2), TA^ΔCP^ (3), TA^ΔCP^ and TB (4–5), TA, TB, and Cβ (6), TA^ΔCP^ and Cβ (7–9), TA and Cβ (10–12) TA^ΔCP^, TB and Cβ (13–15), **(C)** TA and TB (1), TA (2), TA^ΔAC2^ (3), TA, TB and Cβ (4), TA^ΔAC2^ and Cβ (5–8), TA and Cβ (9–11) TA^ΔAC2^ and TB (12), TA^ΔAC2^, TB, and Cβ (13–14), **(D)** TA (1), TA and TB (2), TA^ΔAC4^ (3–4), TA, TB, and Cβ (5), TA^ΔAC4^ and Cβ (6–8), TA and Cβ (9–10), TA^ΔAC4^ and TB (11–12), TA^ΔAC4^, TB and Cβ (13–14). The viral DNA forms are labeled as single-stranded (ss), open-circular (oc), and super-coiled (sc). DNA isolations were performed at 29 dpi and ~10 μg of DNA was resolved in each lane. For each blot a cropped photograph of the genomic DNA bands on the ethidium bromide-stained agarose gel are shown below the blot to show equal loading.

**Figure 3:**
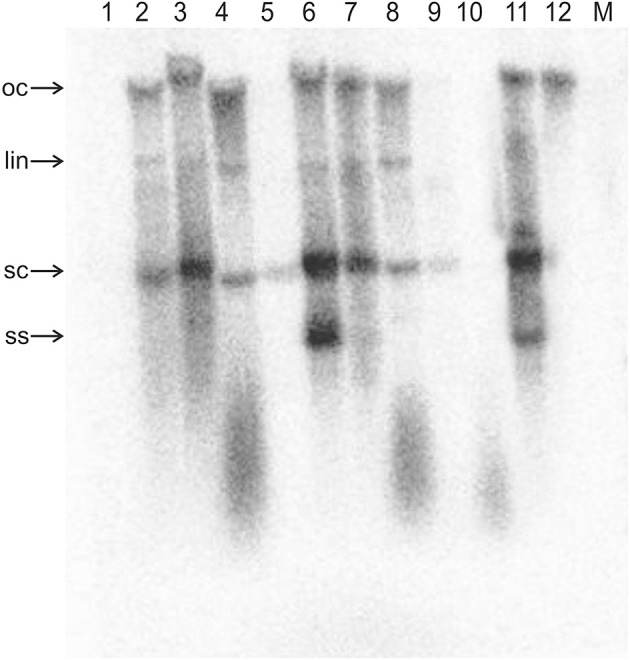
Detection of ToLCNDV DNA B by Southern blotting in inoculated *N. benthamiana* plants. The DNA extracts resolved on the agarose gel were extracted from the leaves of a mock-inoculated plant (M) and from the leaves of plants inoculated with TA^AV2^ (1), TA^ΔAV2^ and TB (2), TA^ΔAV2^, TB, and Cβ (3), TA^ΔAC2^ and TB (4), TA^ΔAC2^, TB, and Cβ (5), TA^ΔCP^ and TB (6), TA^ΔCP^, TB, and Cβ (7), TA^ΔAC4^ and TB (8), TA^ΔAC4^, TB, and Cβ (9), TA (10), and TA and TB (11). The sample loaded in lane 12 consisted of 5 ng of TB plasmid (acc. no. U15017). DNA was extracted from plants at 29 dpi and ~10 μg was resolved in each lane. The blot was exposed to a phosphor screen for 7 days.

Wild type ToLCNDV infection following inoculation with TA and TB induced severe leaf curling, vein thickening, deformed stem and petioles symptoms in *N. benthamiana* plants at 12 days post inoculation (dpi) (Figure 1B). The two DNA components of the virus were readily detected by PCR and Southern blot hybridization (Figures 2A, 3; Table 2). Inoculation of *N. benthamiana* with TA alone did not lead to symptoms. However, in 3 out of 20 plants inoculated, the component could be detected in leaves developing subsequent to inoculation using PCR diagnostics but not by Southern blot hybridization (Table 2; Figure 2A). The titer of TA in plants infected with TA alone was significantly lower (1.786 ng/μg of genomic DNA) than the titer of TA in plants infected with both the components of ToLCNDV (3.730 ng/μg of genomic DNA; Supplementary Table 1).

**Table 2:** Infectivity of ToLCNDV and ToLCNDV DNA A harbouring mutations of the AV2 and CP genes in *N. benthamiana* in the presence and absence of CLCuMuB.

Inoculum^*^	**Infectivity**									**Symptoms^@^**	**Latent period (days)**
	**PCR diagnostics (plants infected/plants inoculated)**						**Southern Blot analysis^†^**				
	**Expt. I**		**Expt. II**		**Expt. III**						
	**TA**	**Cβ**	**TA**	**Cβ**	**TA**	**Cβ**	**TA**	**TB**	**Cβ**		
^#^Mock	0/6	–	0/6	–	0/6	–	(−)	(−)	(−)		
^#^TA	2/11	–	2/12	–	2/12	–	(−)	–	–	NS	–
^#^TA, Cβ	5/8	3/8	5/8	3/8	5/8	3/8	(−)	–	(−)	NS	–
^#^TA, TB	6/6	–	6/6	–	6/6	–	(+)	–	–	ULC, VT, ST	12
^#^TA, TB, Cβ	8/8	4/8	6/6	3/6	6/6	3/6	(+)	–	(−)	ULC, VT, ST	10 (12^$^)
TA^ΔAV2^	0/5	–	0/5	–	1/5	–	(−)	(−)	–	NS	–
TA^ΔAV2^, Cβ	1/5	1/5	2/5	1/5	1/5	1/5	(−)	–	(−)	NS	–
TA^ΔAV2^, TB	5/5	–	5/5	–	5/5	–	(+)	(+)	–	ULC, VT, ST	13-14
TA^ΔAV2^, TB, Cβ	5/5	0/5	4/5	0/5	5/5	1/5	(+)	(+)	(−)	ULC, VT, ST	13
TA^ΔCP^	0/5	–	1/5	–	0/5	–	(−)	–	–	NS	–
TA^ΔCP^, Cβ	4/4	3/4	4/5	2/5	4/5	3/5	(−)	–	(−)	NS	–
TA^ΔCP^, TB	5/5	–	5/5	–	4/5	–	(+)	(+)	–	ULC, VT, ST	13-14
TA^ΔCP^, TB, Cβ	5/5	2/5	4/5	1/5	5/5	2/5	(+)	(+)	(−)	ULC, VT, ST	13

* *ToLCNDV DNA A is denoted as TA, ToLCNDV DNA B as TB and CLCuMuB as Cβ. ToLCNDV DNA A having a mutation of the CP gene is denoted as TA^ΔCP^ and that having a mutation of the AV2 gene as TA^ΔAV2^*.

@ *Symptoms exhibited by plants are shown as upward leaf curling (ULC), stunting (ST), vein thickening (VT), vein yellowing (VY), or no symptoms (NS)*.

$ *For plants in which Cβ was not detected*.

# *Controls in Tables 2, 3 are additive, thus the data for the controls (inoculation with TA, TA/TB, TA/TB/Cβ, and TA/Cβ) is duplicated in Tables 2, 3*.

† *Southern blot hybridization is denoted as either present (+) or absent (−)*.

Co-inoculation of *N. benthamiana* plants with TA and Cβ did not lead to plants exhibiting symptoms. However, by diagnostic PCR, 8 plants (out of 15 inoculated) were shown to contain viral DNA, whereas the betasatellite was detected in only 4 of the TA infected plants (Table 2). Neither component was detected in Southern blotting, showing the level DNAs to be below the threshold for detection by hybridization (Figures 2A, 3A). However, qPCR showed plants co-infected with TA and Cβ to contain a higher titer of TA (2.226 ng/μg of genomic DNA) than plants infected with only TA (1.786 ng/μg of genomic DNA) although this was still less than in TA with TB infections (3.730 ng/μg of genomic DNA) (Supplementary Table 1).

Co-inoculation of *N. benthamiana* plants with TA and TB along with Cβ was as efficient in inducing symptomatic infection as inoculation of both components without the betasatellite (all inoculated plants developed symptoms; Table 2). However, PCR-mediated diagnostics revealed that the betasatellite was present in only 10 out of 20 inoculated plants, whereas TA was detected in all plants by PCR (Table 2). TA and TB but not Cβ were detected in TA, TB, and Cβ infected plants by Southern blot hybridization (Figures 2, 3 and Supplementary Figure 1). The symptoms for *N. benthamiana* plants infected with TA, TB, and Cβ were comparable to the symptoms exhibited by TA/TB infected plants without the betasatellite (Figure 1). However, plants which also contained the betasatellite had a shorter latent period (10 days; the time between inoculation and the first appearance of symptoms) than plants lacking the betasatellite (Table 2). Although the betasatellite could not be detected by Southern blotting, the results of the qPCR analysis showed that the betasatellite DNA level was 1.92 ng (per μg of genomic DNA). Moreover, an enhanced level of TA (5.04 ng/μg of genomic DNA) was evident in presence of TB and betasatellite in comparison to plants infected with TA and TB (3.730 ng/μg of genomic DNA) or TA and Cβ (2.226 ng/μg of genomic DNA; Supplementary Table 1). This indicates an additive effect of the presence of TB and Cβ on TA titer.

*N. benthamiana* plants inoculated with TA bearing a mutation of the AV2 gene (TA^ΔAV2^) failed to develop symptoms and virus could only be detected in one (out of 15) inoculated plants, significantly fewer than for plants inoculated with TA (Supplementary Table 2). However the titer of viral DNA was not significantly lower than for TA infections and in Southern hybridization the component was not detected, indicating that virus DNA levels in this plant were below the detection threshold of Southern blotting.

For plants inoculated with TA^ΔAV2^ and TB, all inoculated plants developed symptoms comparable to wild type ToLCNDV except for the delayed onset of symptoms (13–14 days; Figure 1; Table 2). Surprisingly, the concentration of TA^ΔAV2^ was significantly higher (4.236 ng/μg of genomic DNA; Supplementary Table 1) than for wild type infected plants (3.730 ng/μg of genomic DNA). In this infection, both the viral components (TA and TB) were readily detected by PCR, qPCR and Southern hybridization (Figures 2, 3).

Plants inoculated with TA^ΔAV2^, TB, and Cβ developed symptoms at 13 dpi that could not be distinguished from plants inoculated with just TA^ΔAV2^ and TB (Table 2). PCR-mediated diagnostics showed that the betasatellite was not maintained efficiently (1 out of 15 plants), which is significantly lower than the maintenance of Cβ for TA, TB, and Cβ infections (7 out of 11 plants; Table 2, Supplementary Table 2). Again only viral DNA could be detected by Southern hybridization, not the betasatellite, from total DNA extracted from leaves developing subsequent to inoculation (Figures 2A, 3 and Supplementary Figure 1). Interestingly, a significantly enhanced level of TA^ΔAV2^ (6.337 ng/μg of genomic DNA) was detected in these plants compared to infections of TA^ΔAV2^ with TB (4.236 ng/μg of genomic DNA) and TA^ΔAV2^ with Cβ (2.235 ng/μg of genomic DNA; Supplementary Table 1).

### Effects on infectivity, symptoms and the ability to maintain CLCuMuB of mutation of the CP gene of ToLCNDV

*N. benthamiana* plants inoculated with TA harboring a mutation of the CP gene (TA^ΔCP^) did not develop symptoms (Figure 1) and viral DNA could only be detected by PCR in one plant (of 15 inoculated; Table 2), statistically significantly less than for plants inoculated with TA (3 infected out of 11 inoculated) (Supplementary Table 2). However, no viral DNA was detected in this plant by Southern blotting. The single plant infected with TA^ΔCP^ contained significantly less viral DNA than the plants infected with TA (Supplementary Table 1).

Co-inoculation of TA^ΔCP^ with Cβ to *N. benthamiana* also did not lead to symptoms. However, PCR-mediated diagnostics, but not Southern blotting (Supplementary Figure 1, Table 2), showed the presence of both TA^ΔCP^ (12 plants out of 15 inoculated) and the betasatellite (7 out of 15 plants) in the upper leaves developing subsequent to inoculation. Overall the betasatellite was maintained in fewer plants (15 out of 24) by ToLCNDV DNA A with an intact CP than by DNA A with a mutated CP (8 out of 15), although these numbers are not statistically different (Supplementary Table 2). Also the titer of Cβ was lower in co-infection with TA^ΔCP^ than in co-infection with TA (Supplementary Table 1).

*N. benthamiana* plants inoculated with TA^ΔCP^ and TB developed symptoms that were indistinguishable from those induced by the wild-type virus, although the latent period was somewhat longer (13–14 days compared to 10 days for the wild type virus; Table 2), and the mutation did not affect infectivity—all plants became infected (Table 2). Viral DNA was detected in symptomatic plants by PCR and Southern blotting (Figures 2B, 3). However, TA^ΔCP^/TB infections were associated with a lower DNA A titer than plants infected with TA/TB (Supplementary Table 1). Similarly, inoculation of TA^ΔCP^, TB, and Cβ into *N. benthamiana* plants resulted in symptoms at 13 dpi that were indistinguishable from plants inoculated without the betasatellite. However, in diagnostic PCR only 5 (out of 15 inoculated) plants were shown to contain betasatellite, which indicated that the betasatellite was poorly maintained by the virus having a mutated CP in comparison to the wild type virus (8 out of 9 plants; Table 2), although these numbers are not statistically different (Supplementary Table 2). Of the three components TA and TB, but not the betasatellite, were detected by Southern blotting from total DNA extracted from leaves developing subsequent to inoculation (Figures 2B, 3 and Supplementary Figure 1). Interestingly the presence of either TB or Cβ increased (doubled) the titer of TA^ΔCP^ in co-infected plants (Supplementary Table 1). The inclusion of both TB and Cβ with TA^ΔCP^ had an additive effect, with three times as much TA^ΔCP^ present in infected plants.

### Effects on infectivity, symptoms and the ability to maintain CLCuMuB of mutation of the trap gene of ToLCNDV

**Figure 4:**
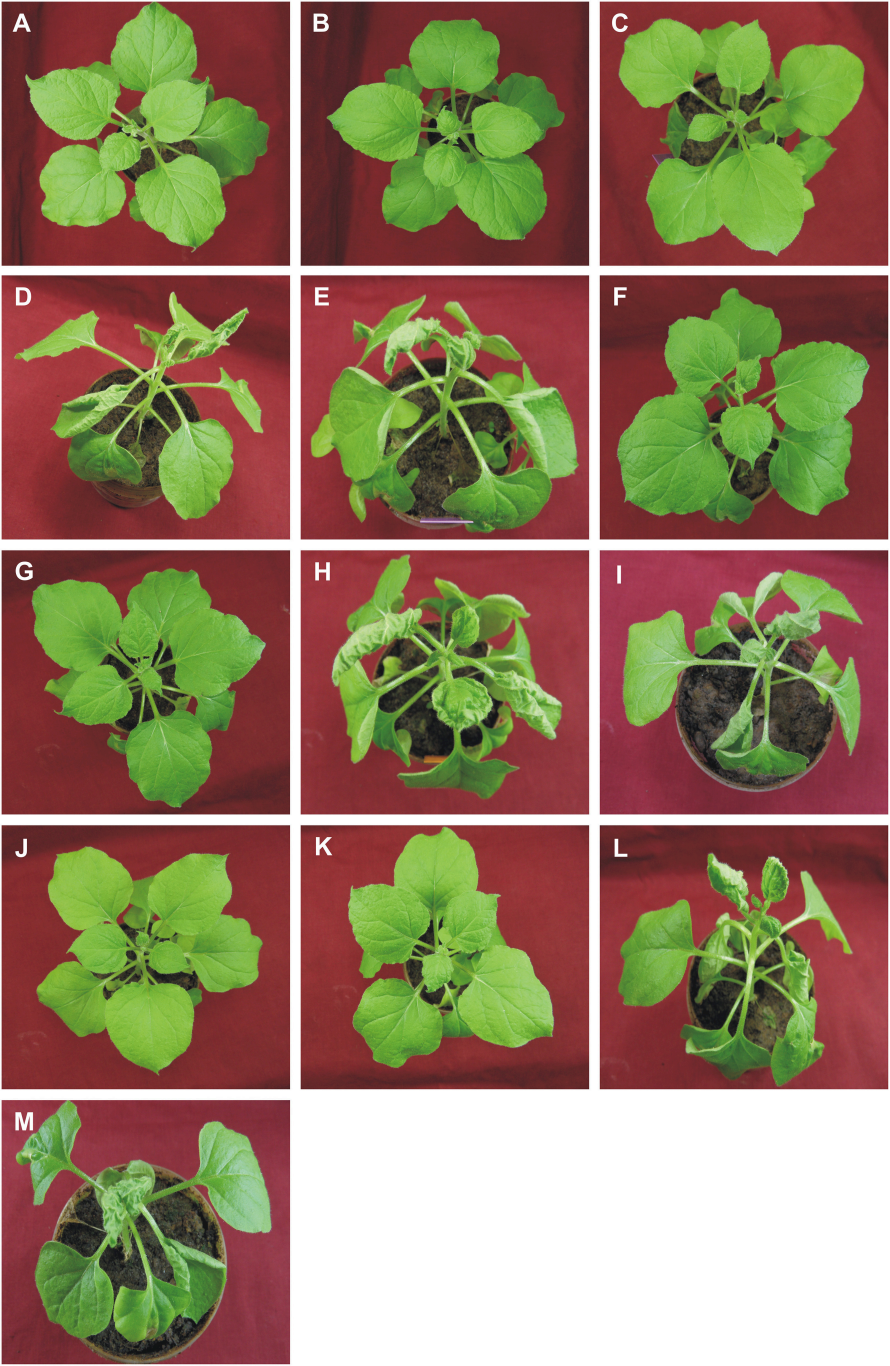
Symptoms in *N. benthamiana* plants following inoculation with ToLCNDV, bearing mutations of the TrAP and AC4 genes, in the presence and absence of CLCuMuB. The plants shown were either not inoculated (healthy; **A**) or inoculated with TA **(B)**, TA and Cβ **(C)**, TA and TB **(D)**, TA, TB, and Cβ **(E)**, TA^ΔC2^ **(F)**, TA^ΔC2^ and Cβ **(G)**, TA^ΔC2^ and TB **(H)**, TA^ΔC2^, TB, and Cβ **(I)**, TA^ΔC4^ **(J)**, TA^ΔC4^ and Cβ **(K)**, TA^ΔC4^ and TB **(L)**, TA^ΔC4^, TB, and Cβ **(M)**. Photographs were taken at 25 dpi.

*N. benthamiana* plants inoculated with TA having a mutation in the TrAP gene (TA^ΔAC2^) failed to develop symptoms (Figure 4). PCR-mediated diagnostics showed that 8 (out of 15 inoculated) plants nevertheless contained viral DNA in tissues distal to the inoculation site (Table 3), statistically significantly more than for wild-type TA (3 out of 10) (Table 3; Supplementary Table 2). By Southern blotting viral DNA could not be detected for TA^ΔAC2^, indicating that the titer of viral DNA was low, below the detection threshold (Figure 2C). qPCR showed the viral titer for TA^ΔAC2^ infected plants not to differ significantly from TA infected plants (Supplementary Table 1).

**Table 3:** Infectivity of ToLCNDV and complementary-sense (TrAP and AC4) gene mutants in *N. benthamiana* in the presence and absence of CLCuMuB.

Inoculum^*^	**Infectivity**									**Symptoms^@^**	**Latent period (days)**
	**PCR diagnostics (plants infected/plants inoculated)**						**Southern Blot analysis^†^**				
	**Expt. I**		**Expt. II**		**Expt. III**						
	**TA**	**Cβ**	**TA**	**Cβ**	**TA**	**Cβ**	**TA**	**TB**	**Cβ**		
^#^Mock	0/6	–	0/6	–	0/6	–	(−)	(−)	(−)		
^#^TA	2/11	–	2/12	–	2/12	–	(−)	–	–	NS	–
^#^TA, Cβ	5/8	3/8	5/8	3/8	5/8	3/8	(−)	–	(−)	NS	–
^#^TA, TB	6/6	–	6/6	–	6/6	–	(+)	–	–	ULC, VT, ST	12
^#^TA, TB, Cβ	8/8	4/8	6/6	3/6	6/6	3/6	(+)	–	(−)	ULC, VT, ST	10 (12^$^)
TA^ΔAC2^	2/5	–	3/5	–	3/5	–	(−)	–	–	NS	–
TA^ΔAC2^, Cβ	2/5	0/5	3/5	0/5	3/5	0/5	(−)	–	(−)	NS	–
TA^ΔAC2^, TB	5/5	–	5/5	–	3/3	–	(+)	(+)	–	ULC, VT, ST	13
TA^ΔAC2^, TB, Cβ	5/5	0/5	5/5	0/5	5/5	0/5	(+)	(+)	(−)	ULC, VT, ST	13
TA^ΔAC4^	2/3	–	3/5	–	2/3	–	(−)	–	–	NS	–
TA^ΔAC4^, Cβ	3/5	0/5	2/3	0/3	4/5	0/5	(−)	–	–	NS	–
TA^ΔAC4^, TB	3/3	–	3/3	–	3/3	–	(+)	(+)	–	ULC, VT, ST	12
TA^ΔAC4^, TB, Cβ	5/5	0/5	5/5	0/5	5/5	1/5	(+)	(+)	(−)	ULC, VT, ST	12

* *ToLCNDV DNA A is denoted as TA, ToLCNDV DNA B as TB, and CLCuMuB as Cβ. ToLCNDV DNA A having a mutation of the TrAP gene is denoted as TA^ΔAC2^ and that having a mutation of the AV4 gene as TA^ΔAC4^*.

@ *Symptoms exhibited by plants are shown as upward leaf curling (ULC), vein thickening (VT), stunting (ST), vein yellowing (VY), mild upward curling of the edges of leaves (EC), or no symptoms (NS)*.

$ *For plants in which Cβ was not detected*.

# *Controls in Tables 2, 3 are additive, thus the data for the controls (inoculation with TA, TA/TB, TA/TB/Cβ, and TA/Cβ) is duplicated in Tables 2, 3*.

† *Southern blot hybridization is denoted as either present (+) or absent (−)*.

Co-inoculation of TA^ΔAC2^ and Cβ also resulted in asymptomatic infection and an equal number of plants (8 out of 15), as for inoculation with TA^ΔAC2^ in the absence of Cβ, showed the presence of viral DNA. Cβ was not detected in TA^ΔAC2^/Cβ inoculated plants by either PCR or Southern hybridization (Table 3; Figure 2C; Supplementary Figure 1). However, qPCR showed the presence of a very low concentration (0.001 ng/μg of genomic DNA) of Cβ and the presence of this did not affect the titer of TA^ΔAC2^ (Supplementary Table 1).

Inoculation of TA^ΔAC2^ with TB to *N. benthamiana* plants induced symptoms that could not be distinguished from TA and TB infected plants, except for a delay in time to appearance of symptoms (13 days rather than 12 days; Table 3). Southern blotting and qPCR detected the mutated virus in infected plants at levels equivalent to plants infected with TA and TB (Figures 2C, 3, 4). As for TA/TB infections, co-infection of TA^ΔAC2^ with TB significantly increased the titer of TA (Supplementary Table 1).

Inoculation of Cβ along with TA^ΔAC2^ and TB did not shorten the latent period (13 dpi). Cβ was not detected in these plants either by PCR or Southern blotting (Table 3; Figures 2C, 3C). The qPCR results showed that betasatellite was nevertheless maintained by the virus bearing a mutation of TrAP but at very low concentration (Supplementary Table 1). Surprisingly, in Southern blotting, a reduced level of TB was detected (Figure 4).

### Effects of mutation of the AC4 gene of ToLCNDV on infectivity, symptoms and the ability to maintain CLCuMuBC

*N. benthamiana* plants inoculated with ToLCNDV DNA A bearing a mutation of the AC4 gene (TA^ΔAC4^) did not develop symptoms of infection. Viral DNA was detected by PCR in 7 out of 11 inoculated plants; statistically significantly more than for plants inoculated with TA (Supplementary Table 2). However, Southern blot hybridization did not detect the virus, indicating that viral DNA levels were low (Figure 2D). qPCR showed the titer of TA^ΔAC4^ to be significantly lower than for plants infected with TA (Supplementary Table 1).

A greater number of asymptomatically infected *N. benthamiana* plants (9 out of 13 inoculated) were detected by PCR upon co-inoculation of TA^ΔAC4^ with Cβ, in comparison to plants inoculated with only TA^ΔAC4^ (7 out of 11; Table 3), although this was not statistically different (Supplementary Table 2). Although Southern blotting failed to detect either component (Figures 2D, 3D), qPCR showed the presence of Cβ to increase the titer of TA^ΔAC4^ in comparison to infections involving only TA^ΔAC4^ (Supplementary Table 1).

All *N. benthamiana* plants inoculated with TA^ΔAC4^ and TB showed symptoms that were qualitatively and quantitatively equivalent to the symptoms induced by wild type TA/TB (Figure 2). This infection was readily detected by PCR, Southern blotting and qPCR. The levels of both TA^ΔAC4^ and TB were comparable to plants infected with wild type TA and TB (Figures 2D, 5; Supplementary Table 1). Co-inoculation of Cβ with TA^ΔAC4^ and TB to *N. benthamiana* plants also resulted in symptoms typical of a wild type TA/TB infection (Figure 2). All the inoculated plants (9 out of 9) were symptomatic, but betasatellite was detected in only one plant (Table 3). Viral DNA, but not the betasatellite, was detected by Southern blotting (Figures 2D, 3D). However, Southern blotting showed a possible reduced accumulation of TB (Figure 3). This was not supported by the qPCR data which suggested a reduced titer of TA. This apparently contradictory result possibly is due to only a single plant having been analyzed. For inoculation of TA^ΔAC4^ with Cβ in no plants was Cβ maintained. These inoculated plants thus resemble TA^ΔAC4^ infected plants in all respects.

## Discussion

Although more commonly associated with monopartite begomoviruses, betasatellites are being reported infrequently, but increasingly, with bipartite begomoviruses (Ilyas et al., 2010; Jyothsna et al., 2013; Zaidi et al., 2016). The effects of betasatellites on bipartite begomovirus infections has not been investigated in any detail although one study has suggested that the interaction between a bipartite begomovirus and a betasatellite would not be stable. The study described here was intended to investigate the effects of a betasatellite on the infection of a bipartite begomovirus and to determine the viral requirements for maintenance of a betasatellite by a bipartite begomovirus.

ToLCNDV is highly infectious to *N. benthamiana* but also has the ability to trans-replicate and maintain CLCuMuB (Cβ). However, the betasatellite was maintained in only about 50% of inoculated plants. No change in symptoms was noted, although the presence of the betasatellite reduced the latent period for the infection. Inoculation of plants with only the TA did not lead to symptomatic infection, although the virus component could be detected distal to the inoculation site in a small number of plants. This is consistent with earlier findings which have shown that the DNA A component of bipartite begomoviruses may spread in plants, without inducing symptoms and at low DNA titer, in the absence of DNA B (Klinkenberg and Stanley, 1990; Evans and Jeske, 1993b; Briddon and Markham, 2001; Saunders et al., 2002; Fontenelle et al., 2007). Inoculation of TA with Cβ partially complemented missing DNA B functions (more plants showing the presence of TA than in plants inoculated with TA in the absence of the betasatellite) but did not lead to symptomatic infection. This contrasts with previous studies which showed that TA and Cβ induced symptoms in tomato and mild, but transient, symptoms in cotton (Saeed et al., 2007; Saeed, 2010). The reason for the difference is unclear but may be due to the different methods of inoculation used (biolistic inoculation for the earlier studies and the *Agrobacterium*-mediated method used here). It has previously been noted that for *Agrobacterium*-mediated inoculation some host/inoculum combinations (particularly for begomoviruses associated with betasatellites) are problematic. For example, no infectivity of tomato was achieved by *Agrobacterium*-mediated inoculation of ToLCNDV DNA A with CLCuMuB, even though this combination was infectious to tomato by biolistic inoculation (Saeed, 2008). Similarly, CLCuMuV with CLCuMuB can be biolistically inoculated to cotton (Briddon et al., 2001) although cotton has so far proven recalcitrant to inoculation with these components using *Agrobacterium* (unpublished results).

The symptoms induced by ToLCNDV DNA A bearing a mutation of the AV2 gene (TA^ΔAV2^), in the presence of the DNA B, were qualitatively the same as the symptoms induced by the wild type virus in *N. benthamiana* but attenuated with a longer latent period. However, the mutation did not affect infectivity, with all plants showing symptoms. This is consistent with previous findings for bipartite begomoviruses (Padidam et al., 1996; Rouhibakhsh et al., 2011). In the absence of the DNA B, TA^ΔAV2^ infections were non-symptomatic and the mutation significantly reduced the numbers of plants infected compared to plants inoculated with only TA. In the presence of TB, Cβ was maintained poorly both by TA^ΔAV2^ and wild type DNA A. However, co-inoculation of TA^ΔAV2^ with Cβ led to more plants becoming infected in comparison to TA^ΔAV2^ alone, showing that the betasatellite can, at least to some degree, complement missing AV2 functions. The role of AV2 for OW bipartite begomoviruses remains unclear, since movement functions are provided by NSP and MP encoded by DNA B. However, involvement of AV2 has been shown in cell-to-cell trafficking and suppression of gene silencing in bipartite begomoviruses (Padidam et al., 1996; Rothenstein et al., 2007; Chowda-Reddy et al., 2008). Here levels of viral DNA were the same for the AV2 mutant and the wild type virus. This contrasts with previous findings, where a reduced level of viral DNA was observed in *N. benthamiana* for AV2 mutants (Padidam et al., 1996; Rouhibakhsh et al., 2011) and that mutation of the AV2 affected CP expression, although the precise mechanism was not defined (Bull et al., 2007). The results also show that in the presence of the DNA B, AV2 is important for maintenance of the betasatellite but not in the absence of the DNA B. This difference may possibly be explained by the size-specific binding of DNA by the NSP and MP proteins encoded on DNA B. For a NW bipartite begomovirus both NSP and MP have been shown to preferentially bind DNA that is larger than the DNA of betasatellites (Rojas et al., 1998). It is thus possible that a betasatellite can only be efficiently maintained when a second protein, AV2, provides movement functions. In the absence of the DNA B the betasatellite would move by the same mechanism, likely involving the CP, and at the same rate as the DNA A.

Begomoviruses native to the NW lack the (A)V2 gene, leading to the suggestion that this may be the reason for the apparent under representation of monopartite begomoviruses in this region; only a single monopartite begomovirus having been so far identified in the NW (Melgarejo et al., 2013; Sánchez-Campos et al., 2013). Nawaz-ul-Rehman et al. (2009) showed that the NW bipartite begomovirus *Cabbage leaf curl virus* could maintain CLCuMuBin the presence of the DNA B but not in its absence (Nawaz-ul-Rehman et al., 2009). This contrasts with the ability of CLCuMuB to complement the DNA B of OW begomoviruses (Saeed et al., 2007) and the ability of CLCuMuB to complement (at least for infectivity) the AV2 mutation shown here. This is suggestive of more differences between NW and OW begomoviruses rather than just the absence of the AV2 gene. Possibly the absence of the AV2 gene in NW viruses has led the DNA A to become more reliant on DNA B functions and this may be reflected in the distinct, and conserved amino acid differences of, for example, the CP of viruses from these two regions (Ha et al., 2006).

Inoculation of *N. benthamiana* plants with ToLCNDV DNA A bearing a mutation of the CP (TA^ΔCP^) and DNA B resulted in infections with symptoms comparable to infections of the wild type virus. The only difference was that the latent period was longer for the mutated virus. This is in agreement with earlier studies which showed that bipartite begomoviruses lacking the CP are infectious and the longer latent period suggested that the CP is required for fast long-distance movement of virus in the phloem (Brough et al., 1988; Etessami et al., 1989; Padidam et al., 1996; Briddon and Markham, 2001; Rojas et al., 2001; Rouhibakhsh et al., 2011). Inoculation of plants with TA^ΔCP^, but without the DNA B, resulted in asymptomatic infections, but with significantly fewer plants infected than for inoculation with TA. This again is consistent with the idea that the CP is important for movement, likely required to protect viral ssDNA in the phloem. Co-inoculation of Cβ with TA^ΔCP^ also resulted in plants that were asymptomatically infected. Far more plants were infected than for inoculations with only TA^ΔCP^, consistent with the idea that the βC1 encoded by betasatellites is involved in virus movement and may complement missing DNA B functions (Saeed et al., 2007). Although more plants were shown to maintain the betasatellite than for inoculations of TA with Cβ, this was not statistically significant. This could indicate that there is an antagonism between the betasatellite and the CP. Since betasatellites only encode a single product, βC1 (Saunders et al., 2004), the antagonism would likely be between CP and βC1. Working on the hypothesis that the CP is required for protection of viral DNA during movement, particularly in the phloem, it is possible that βC1 similarly protects the viral (and betasatellite) DNA during movement. This is consistent with the finding that βC1 binds DNA (Cui et al., 2005). It is also consistent with the idea that βC1 facilitates virus movement from the site of inoculation to the phloem (Saeed et al., 2007) and/or facilitates cell entry (re-infection) distal to the inoculation site after movement of the virus in the phloem. Kumar et al. (2006) showed interaction of the CP of *Bhendi yellow vein mosaic virus* and the βC1 of Bhendi yellow vein betasatellite. The CP of geminiviruses plays a direct role in viral nuclear entry by associating with the viral ssDNA, protecting it from nucleolytic degradation, and supplying it with nuclear localization signals (Palanichelvam et al., 1998). For bipartite begomoviruses this function is likely masked in the presence of DNA B, which encodes a protein specifically tasked with shuttling viral DNA in and out of the nucleus—the NSP (Gafni and Epel, 2002). However, nuclear localization provided by the CP is likely important early during infection, following delivery of virus particles by insect vectors, when expression of viral proteins has yet to occur.

In contrast to the findings with several other bipartite begomoviruses (Brough et al., 1988; Etessami et al., 1991; Evans and Jeske, 1993a), mutation of the TrAP gene of ToLCNDV did not prevent infection and the mutant virus (in the presence of the DNA B) induced wild-type symptoms in *N. benthamiana*. The reason for the difference between ToLCNDV and the other viruses examined is unclear. Two of the studies were on New World begomoviruses, *Abutilon mosaic virus* (Evans and Jeske, 1993a) and *Tomato golden mosaic virus* (Brough et al., 1988), and produced double mutations of the TrAP and REn genes, rather than single mutations. This might have significantly disabled the virus since REn enhances DNA replication by interacting with Rep and various host factors (Settlage et al., 2005). The third study mutated *African cassava mosaic virus*, an OW begomovirus. Possibly this virus is not well adapted to plants of the *Solanaceae*.

Surprisingly, mutation of the TrAP gene more than doubled the numbers of plants in which there was independent spread of the component. The reason for this is unclear. Possibly *N. benthamiana* has a resistance which targets the TrAP of ToLCNDV. However, this would seem unlikely since expression of avirulence determinants is usually associated with a hypersensitive response (necrosis) in the resistant host, which is not the case here (even when overexpressed from a *Potato virus X* vector or under the control of the *Cauliflower mosaic virus* 35S promoter; data not shown). In fact the TrAP of ToLCNDV has been shown to overcome the hypersensitive cell death induced by other virus gene products (Hussain et al., 2007).

In both the presence and absence of the DNA B, the DNA A bearing a mutation of TrAP (TA^ΔAC2^) did not maintain CLCuMuB. This indicates that, as was found for the monopartite begomovirus *Cotton leaf curl Kokhran virus* (CLCuKoV) (Iqbal et al., 2012), TrAP is important for the maintenenace of a betasatellite. TrAP performs multiple functions. It may act as a transcription factor to up-regulate expression of late (virion-sense) genes (Sunter and Bisaro, 1991; Gopal et al., 2007), modulates host gene expression including micro RNA genes (Trinks et al., 2005; Amin et al., 2011a), can be a pathogenicity factor (Van Wezel et al., 2001; Matić et al., 2016), a suppressor of transcriptional and post-transcriptional gene silencing (van Wezel et al., 2003; Buchmann et al., 2009; Jackel et al., 2015; Kumar et al., 2015), may delay virus infection (Shen et al., 2014; Krenz et al., 2015), may counter programmed cell death (Hussain et al., 2007; Mubin et al., 2010), conditions a virus non-specific enhanced-susceptibility phenotype (Sunter et al., 2001), suppresses jasmonate-mediated defense (Rosas-Díaz et al., 2016), and inactivates the SNF1-related kinase by interacting with it and adenosine kinase (Hao et al., 2003). Clearly TrAP is an important protein for ToLCNDV being involved in control of gene expression and numerous interactions with the host plant. Loss of any one of these could be the reason for the lack of maintenance of the betasatellite when TrAP is mutated and further studies will be required to determine which of the TrAP functions are required for betasatellite maintenance.

Mutation of the AC4 gene of ToLCNDV had no effect on the infectivity or symptoms of the virus in the presence of the DNA B—all plants were infected/symptomatic. This is consistent with previous studies that mutated the AC4 gene of bipartite begomoviruses and indicates that the product of AC4 is not required for either infectivity or the induction of symptoms (Etessami et al., 1991; Hoogstraten et al., 1996; Pooma and Petty, 1996; Fontenelle et al., 2007). However, in the absence of the DNA B, the DNA A component with the AC4 mutation was significantly more infectious to *N. benthamiana* than the wild type. The reason for this is unclear. The precise function of the (A)C4 product remains uncertain and may differ between monopartite and bipartite begomoviruses. For some begomoviruses it is a pathogenicity/symptom determinant, a suppressor of post-transcriptional gene silencing and interferes with micro RNA expression of the host (Vanitharani et al., 2004; Gopal et al., 2007; Saeed et al., 2008; Amin et al., 2011a). For monopartite begomoviruses the C4 is implicated in virus movement (Rojas et al., 2001) and preventing DNA methylation (suppressing transcriptional gene silencing) (Rodríguez-Negrete et al., 2013). Although the NSP of ToLCNDV has been shown to be an avirulence determinant in tobacco (Hussain et al., 2007), it is possible that in *N. benthamiana* the AC4 product is a weak avirulence determinant, which is masked in the presence of the DNA B, and mutation of the gene relieves the virus from the effects of a host resistance leading to enhanced levels of infectivity.

Although for wild type ToLCNDV (TA with TB) infections with Cβ, the betasatellite was maintained in about 50% of plants, mutation of the AC4 gene significantly reduced the numbers of plants in which the betasatellite was maintained. In the absence of the TB, the AC4 mutant virus did not maintain the betasatellite. This indicates that for ToLCNDV the AC4 protein is important in the maintenance of the betasatellite in both the presence and absence of the DNA B. These results contrasts with the results for the monopartite begomovirus CLCuKoV, for which the C4 was found not to be important for the maintenance of a betasatellite in *N. benthamiana*, highlighting possible differences between mono- and bipartite begomoviruses (Iqbal et al., 2012). Nevertheless, it is evident that for the bipartite begomovirus ToLCNDV the AC4 protein plays a part in the maintenance of a betasatellite. However, the results are consistent with the AC4 of ToLCNDV having a role in virus movement, as discussed above for monopartite begomoviruses. The finding that the AC4 protein of some bipartite begomoviruses have suppressor of RNAi activity (Vanitharani et al., 2004), although this possibility has not been investigated for ToLCNDV yet, might suggest that, rather than being a classical movement protein, it instead overcomes a host RNAi-based resistance to movement.

The work conducted here has shown that the TrAP is required for maintenance of the betasatellite and the AC4 protein is important for maintenance, particularly in the absence of the DNA B. The AV2 was shown to be important for betasatellite maintenance, but only in the presence of the DNA B. In contrast, the CP appeared to interfere with the maintenance of the betasatellite in the absence of the DNA B. These findings are in general agreement with the earlier study of requirements for maintenance of a betasatellite by a monopartite begomoviruses except for the C4, which was shown not to be important for betasatellite maintenance by a monopartite begomovirus (Iqbal et al., 2012). The reason for the difference is unclear but could indicate virus specific differences with respect to overcoming host RNAi based defenses. For example, for the related bipartite begomoviruses *African cassava mosaic virus* and the Cameroon strain of *East African cassava mosaic virus* (formerly East African cassava mosaic Cameroon virus) the TrAP and AC4 protein play different roles in suppression of post-transcriptional gene silencing (Vanitharani et al., 2004). It will thus be necessary to examine further viruses to assess whether the results obtained here with ToLCNDV, and earlier with CLCuKoV, are typical of all bipartite and monopartite begomoviruses.

Although the presence of the betasatellite did not appear to significantly enhance symptoms of ToLCNDV infections in *N. benthamiana*, which contrasts with the results for another isolate of ToLCNDV (Jyothsna et al., 2013), the viral DNA A levels were significantly increased in the presence of the satellite. The prominent increase in DNA B levels and decrease in betasatellite levels reported by Jyothsna et al. (2013) were not seen here. Significant falls in betasatellite DNA titer was only detected for infections with the TrAP mutant virus. Thus a betasatellite can enhance bipartite begomovirus infections although it remains unclear whether this interaction is stable, whether the betasatellite will be maintained long term.

Having investigated the interactions between a betasatellite and both monopartite and bipartite helper begomoviruses, it will be interesting to investigate the effects of mutations of virus-encoded genes on the other geminivirus-associated satellites, the alphasatellites and the betasatellite-derived deltasatellites (Dry et al., 1997; Nawaz-ul-Rehman et al., 2010; Idris et al., 2011; Fiallo-Olivé et al., 2012, 2016; Hassan et al., 2016; Lozano et al., 2016). Both types of molecules appear to lack a strong selection mechanism for their maintenance by begomoviruses (such as that provided, in some hosts, by βC1 for betasatellites) and alphasatellites differ from betasatellites in being capable of autonomous replication (Mansoor et al., 1999). The interactions of these satellites with begomoviruses will be the focus of future studies.

## Conclusions

The interaction of a bipartite begomovirus with a betasatellite was found to be more complex than just transreplication by the virus. The study here showed TrAP to be essential for maintenance of a betasatellite and AV2 to be important only in the presence of the DNA B. AC4 was found to be important for the maintenance of the betasatellite in the presence of DNA B but essential in the absence of the DNA B. Rather than being required for maintenance, the CP was shown to possibly interfere with maintenance of the betasatellite. These results differ from those obtained for an analysis of the maintenance by a monopartite begomovirus of a betasatellite and show that the interaction of betasatellites with begomoviruses is complex. Overall the apparent conflict between CP and the betasatellite suggests that the interaction (bipartite begomovirus [DNA A, DNA B] and betasatellite) will be unstable and will not lead to supervirulent tripartite viruses. Nevertheless, the presence of both the DNA B and betasatellite significantly increases DNA A titer. Since such infections can occur with some regularity in the field, due to co-infection of a bipartite begomovirus and a betasatellite-associated monopartite begomovirus, they can nevertheless cause significant additional losses to crops.

## Author contributions

ZI performed the majority of the experimental work. MS conducted the qPCR and some of the Southern blot hybridization analyses. RB conceived the study and supervised the work. ZI and IA prepared the first draft of the manuscript which was edited by RB and SM. The final manuscript was read and approved by all authors.

### Conflict of interest statement

The authors declare that the research was conducted in the absence of any commercial or financial relationships that could be construed as a potential conflict of interest.

## References

[B1] Adams M. J. King A. M. Q. Carstens E. B. (2013). Ratification vote on taxonomic proposals to the international committee on taxonomy of viruses. Arch. Virol. 158, 2023–2030. 10.1007/s00705-013-1688-5 23580178

[B2] Akhter A. Qazi J. Saeed M. Mansoor S. (2009). A severe leaf curl disease on chilies in Pakistan is associated with multiple begomovirus components. Plant Dis. 93:962 10.1094/PDIS-93-9-0962B 30754557

[B3] Amin I. Ilyas M. Mansoor S. Briddon R. W. Saeed M. (2010). Role of DNA satellites in geminiviral disease complexes, in Emerging Geminiviral Diseases and their Management, eds Sharma P. Gaur R. K. Ikegami M. (New York, NY: Nova Science Publishers Inc.), 209–234.

[B4] Amin I. Patil B. L. Briddon R. W. Mansoor S. Fauquet C. M. (2011a). A common set of developmental miRNAs are upregulated in *Nicotiana benthamiana* by diverse begomoviruses. Virol. J. 8:143. 10.1186/1743-422X-8-143 21447165 PMC3072929

[B5] Amin I. Patil B. L. Briddon R. W. Mansoor S. Fauquet C. M. (2011b). Comparison of phenotypes produced in response to transient expression of genes encoded by four distinct begomoviruses in *Nicotiana benthamiana* and their correlation with the levels of developmental miRNAs. Virol. J. 8:238. 10.1186/1743-422X-8-238 21592402 PMC3166278

[B6] Anwar S. (2017). Distinct association of an alphasatellite and a betasatellite with tomato leaf curl New Delhi virus in field-infected cucurbit. J. Gen. Plant Pathol. 83, 185–188 10.1007/s10327-017-0709-8

[B7] Argüello-Astorga G. R. Guevara-González L. R. Herrera-Estrella L. R. Rivera-Bustamante R. F. (1994). Geminivirus replication origins have a group-specific organization of iterative elements: a model for replication. Virology 203, 90–100. 10.1006/viro.1994.1458 8093156

[B8] Baliji S. Lacatus G. Sunter G. (2010). The interaction between geminivirus pathogenicity proteins and adenosine kinase leads to increased expression of primary cytokinin-responsive genes. Virology 402, 238–247. 10.1016/j.virol.2010.03.023 20399479 PMC2876732

[B9] Briddon R. W. Mansoor S. (2008). Beta ssDNA satellites, in Encyclopedia of Virology, eds Mahy B. W. J. van Regenmortel M. H. V. (Oxford: Academic Press), 314–321.

[B10] Briddon R. W. Markham P. G. (2001). Complementation of bipartite begomovirus movement functions by topocuviruses and curtoviruses. Arch. Virol. 146, 1811–1819. 10.1007/s007050170067 11699966

[B11] Briddon R. W. Bull S. E. Amin I. Idris A. M. Mansoor S. Bedford I. D.. (2003). Diversity of DNA β, a satellite molecule associated with some monopartite begomoviruses. Virology 312, 106–121. 10.1016/S0042-6822(03)00200-9 12890625

[B12] Briddon R. W. Mansoor S. Bedford I. D. Pinner M. S. Markham P. G. (2000). Clones of cotton leaf curl geminivirus induce symptoms atypical of cotton leaf curl disease. Virus Genes 20, 17–24. 10.1023/A:1008151921937 10766303

[B13] Briddon R. W. Mansoor S. Bedford I. D. Pinner M. S. Saunders K. Stanley J.. (2001). Identification of DNA components required for induction of cotton leaf curl disease. Virology 285, 234–243. 10.1006/viro.2001.0949 11437658

[B14] Brough C. L. Hayes R. J. Morgan A. J. Coutts R. H. A. Buck K. W. (1988). Effects of mutagenesis *in vitro* on the ability of cloned tomato golden mosaic virus DNA to infect *Nicotiana benthamiana* plants. J. Gen. Virol. 69, 503–514. 10.1099/0022-1317-69-3-503

[B15] Brown J. K. Fauquet C. M. Briddon R. W. Zerbini M. Moriones E. Navas-Castillo J. (2012). Geminiviridae. London; San Diego, CA: Waltham; Associated Press; Elsevier Inc.

[B16] Buchmann R. C. Asad S. Wolf J. N. Mohannath G. Bisaro D. M. (2009). Geminivirus AL2 and L2 proteins suppress transcriptional gene silencing and cause genome-wide reductions in cytosine methylation. J. Virol. 83, 5005–5013. 10.1128/JVI.01771-08 19279102 PMC2682068

[B17] Bull S. E. Briddon R. W. Sserubombwe W. S. Ngugi K. Markham P. G. Stanley J. (2007). Infectivity, pseudorecombination and mutagenesis of *Kenyan cassava* mosaic begomoviruses. J. Gen. Virol. 88, 1624–1633. 10.1099/vir.0.82662-0 17412996

[B18] Castillo-González C. Liu X. Huang C. Zhao C. Ma Z. Hu T.. (2015). Geminivirus-encoded TrAP suppressor inhibits the histone methyltransferase SUVH4/KYP to counter host defense. eLife 4:e06671. 10.7554/eLife.06671 26344546 PMC4606454

[B19] Cheng X. Wang X. Wu J. Briddon R. W. Zhou X. (2011). βC1 encoded by tomato yellow leaf curl China betasatellite forms multimeric complexes *in vitro* and *in vivo*. Virology 409, 156–162. 10.1016/j.virol.2010.10.007 21035158

[B20] Chowda-Reddy R. V. Achenjang F. Felton C. Etarock M. T. Anangfac M.-T. Nugent P.. (2008). Role of a geminivirus AV2 protein putative protein kinase C motif on subcellular localization and pathogenicity. Virus Res. 135, 115–124. 10.1016/j.virusres.2008.02.014 18405995

[B21] Cui X. Li G. Wang D. Hu D. Zhou X. (2005). A begomovirus DNAβ-encoded protein binds DNA, functions as a suppressor of RNA silencing, and targets the cell nucleus. J. Virol. 79, 10764–10775. 10.1128/JVI.79.16.10764-10775.2005 16051868 PMC1182626

[B22] Dalakouras A. Moser M. Zwiebel M. Krczal G. Hell R. Wassenegger M. (2009). A hairpin RNA construct residing in an intron efficiently triggered RNA-directed DNA methylation in tobacco. Plant J. 60, 840–851. 10.1111/j.1365-313X.2009.04003.x 19702668

[B23] Doyle J. J. Doyle J. L. (1990). Isolation of plant DNA from fresh tissue. Focus 12, 13–15.

[B24] Dry I. Krake L. R. Rigden J. E. Rezaian M. A. (1997). A novel subviral agent associated with a geminivirus: the first report of a DNA satellite. Proc. Natl. Acad. Sci. U.S.A. 94, 7088–7093. 10.1073/pnas.94.13.7088 9192696 PMC21289

[B25] Etessami P. Saunders K. Watts J. Stanley J. (1991). Mutational analysis of complementary-sense genes of African cassava mosaic virus DNA A. J. Gen. Virol. 72, 1005–1012. 10.1099/0022-1317-72-5-1005 2033385

[B26] Etessami P. Watts J. Stanley J. (1989). Size reversion of African cassava mosaic virus coat protein gene deletion mutants during infection of *Nicotiana benthamiana*. J. Gen. Virol. 70, 277–289. 10.1099/0022-1317-70-2-277 2732690

[B27] Evans D. Jeske H. (1993a). Complementation and recombination between mutants of complementary sense genes of DNA A of Abutilon mosaic virus. Virology 197, 492–496. 10.1006/viro.1993.1619 8212592

[B28] Evans D. Jeske H. (1993b). DNA B facilitates, but is not essential for, the spread of Abutilon mosaic virus in agroinoculated *Nicotiana benthamiana*. Virology 194, 752–757. 10.1006/viro.1993.1316 8503185

[B29] Fiallo-Olivé E. Martínez-Zubiaur Y. Moriones E. Navas-Castillo J. (2012). A novel class of DNA satellites associated with new world begomoviruses. Virology 426, 1–6. 10.1016/j.virol.2012.01.024 22330203

[B30] Fiallo-Olivé E. Tovar R. Navas-Castillo J. (2016). Deciphering the biology of deltasatellites from the new world: maintenance by new world begomoviruses and whitefly transmission. New Phytol. 212, 680–692. 10.1111/nph.14071 27400152

[B31] Fontenelle M. R. Luz D. F. Gomes A. P. S. Florentino L. H. Zerbini F. M. Fontes E. P. B. (2007). Functional analysis of the naturally recombinant DNA-A of the bipartite begomovirus tomato chlorotic mottle virus. Virus Res. 126, 262–267. 10.1016/j.virusres.2007.02.009 17367887

[B32] Gafni Y. Epel B. L. (2002). The role of host and viral proteins in intra- and inter-cellular trafficking of geminiviruses. Physiol. Mol. Plant Pathol. 60, 231–241. 10.1006/pmpp.2002.0402

[B33] Gladfelter H. J. Eagle P. A. Fontes E. P. B. Batts L. Hanley-Bowdoin L. (1997). Two domains of the AL1 protein mediate geminivirus origin recognition. Virology 239, 186–197. 10.1006/viro.1997.8869 9426458

[B34] Gopal P. Kumar P. Sinilal B. Jose J. Kasin Yadunandam A. Usha R. (2007). Differential roles of C4 and βC1 in mediating suppression of post-transcriptional gene silencing: evidence for transactivation by the C2 of Bhendi yellow vein mosaic virus, a monopartite begomovirus. Virus Res. 123, 9–18. 10.1016/j.virusres.2006.07.014 16949698

[B35] Ha C. Coombs S. Revill P. Harding R. Vu M. Dale J. (2006). Corchorus yellow vein virus, a new world geminivirus from the old World. J. Gen. Virol. 87, 997–1003. 10.1099/vir.0.81631-0 16528050

[B36] Haider M. S. Tahir M. Latif S. Briddon R. W. (2006). First report of tomato leaf curl New Delhi virus infecting *Eclipta prostrata* in Pakistan. Plant Pathol. 53:285 10.1111/j.1365-3059.2005.01278.x

[B37] Hanley-Bowdoin L. Settlage S. B. Robertson D. (2004). Reprogramming plant gene expression: a prerequisite to geminivirus DNA replication. Mol. Plant Pathol. 5, 149–156. 10.1111/j.1364-3703.2004.00214.x 20565592

[B38] Hanley-Bowdoin L. Settlage S. B. Orozco B. M. Nagar S. Robertson D. (1999). Geminviruses: models for plant DNA replication, transcription, and cell cycle regulation. Crit. Rev. Plant Sci. 18, 71–106. 10.1080/07352689991309162 10821479

[B39] Hao L. Wang H. Sunter G. Bisaro D. M. (2003). Geminivirus AL2 and L2 proteins interact with and inactivate SNF1 kinase. Plant Cell 15, 1034–1048. 10.1105/tpc.009530 12671096 PMC152347

[B40] Hassan I. Orílio A. F. Fiallo-Olivé E. Briddon R. W. Navas-Castillo J. (2016). Infectivity, effects on helper viruses and whitefly transmission of the deltasatellites associated with sweepoviruses (genus Begomovirus, family Geminiviridae). Sci. Rep. 6:30204. 10.1038/srep30204 27453359 PMC4958995

[B41] Hellens R. P. Edwards E. A. Leyland N. R. Bean S. Mullineaux P. M. (2000). pGreen: a versatile and flexible binary Ti vector for Agrobacterium-mediated plant transformation. Plant Mol. Biol. 42, 819–832. 10.1023/A:1006496308160 10890530

[B42] Hoogstraten R. A. Hanosen S. F. Maxwell D. P. (1996). Mutational analysis of the putative nicking motif in the replication-associated protein (AC1) of bean golden mosaic virus. Mol. Plant Microbe Interact. 9, 594–599. 10.1094/MPMI-9-0594 8810074

[B43] Hussain M. Mansoor S. Iram S. Fatima A. N. Zafar Y. (2005). The nuclear shuttle protein of tomato leaf curl New Delhi virus is a pathogenicity determinant. J. Virol. 79, 4434–4439. 10.1128/JVI.79.7.4434-4439.2005 15767443 PMC1061533

[B44] Hussain M. Mansoor S. Iram S. Zafar Y. Briddon R. W. (2004). First report of tomato leaf curl New Delhi virus affecting chilli pepper in Pakistan. Plant Pathol. 54:794 10.1111/j.1365-3059.2004.01073.x

[B45] Hussain M. Mansoor S. Iram S. Zafar Y. Briddon R.W. (2007). The hypersensitive response to tomato leaf curl New Delhi virus nuclear shuttle protein is inhibited by transcriptional activator protein. Mol. Plant-Microbe Interact. 20, 1581–1588. 10.1094/MPMI-20-12-1581 17990965

[B46] Idris A. M. Shahid M. S. Briddon R. W. Khan A. J. Zhu J.-K. Brown J. K. (2011). An unusual alphasatellite associated with monopartite begomoviruses attenuates symptoms and reduces betasatellite accumulation. J. Gen. Virol. 92, 706–717. 10.1099/vir.0.025288-0 21084498

[B47] Ilyas M. Qazi J. Mansoor S. Briddon R. W. (2010). Genetic diversity and phylogeography of begomoviruses infecting legumes in Pakistan. J. Gen. Virol. 91, 2091–2101. 10.1099/vir.0.020404-0 20375225

[B48] Iqbal Z. (2013). Analysis of the Virus-Encoded Genes Required for the Maintenance of Betasatellites by Geminiviruses, Department of Biotechnology (NIBGE). Islamabad: Pakistan Institute of Engineering and Applied Sciences.

[B49] Iqbal Z. Sattar M. N. Kvarnheden A. Mansoor S. Briddon R. W. (2012). Effects of the mutation of selected genes of Cotton leaf curl Kokhran virus on infectivity, symptoms and the maintenance of cotton leaf curl multan betasatellite. Virus Res. 169, 107–116. 10.1016/j.virusres.2012.07.016 22871297

[B50] Ito T. Sharma P. Kittipakorn K. Ikegami M. (2008). Complete nucleotide sequence of a new isolate of tomato leaf curl New Delhi virus infecting cucumber, bottle gourd and muskmelon in Thailand. Arch. Virol. 153, 611–613. 10.1007/s00705-007-0029-y 18193155

[B51] Jackel J. N. Buchmann R. C. Singhal U. Bisaro D. M. (2015). Analysis of geminivirus AL2 and L2 proteins reveals a novel AL2 silencing suppressor activity. J. Virol. 89, 3176–3187. 10.1128/JVI.02625-14 25552721 PMC4337558

[B52] Juarez M. A. Tovar R. Fiallo-Olivé E. Aranda M. A. Gosálvez B. Castillo P. (2014). First detection of tomato leaf curl New Delhi virus infecting zucchini in Spain. Plant Dis. 98:857 10.1094/PDIS-10-13-1050-PDN 30708660

[B53] Jyothsna P. Haq Q. M. I. Singh P. Sumiya K. V. Praveen S. Rawat R.. (2013). Infection of tomato leaf curl New Delhi virus (ToLCNDV), a bipartite begomovirus with betasatellites, results in enhanced level of helper virus components and antagonistic interaction between DNA B and betasatellites. Appl. Microbiol. Biotechnol. 97, 5457–5471. 10.1007/s00253-012-4685-9 23306645

[B54] Klinkenberg F. A. Stanley J. (1990). Encapsidation and spread of African cassava mosaic virus DNA A in the absence of DNA B when agroinoculated to *Nicotiana benthamiana*. J. Gen. Virol. 71, 1409–1412. 10.1099/0022-1317-71-6-1409

[B55] Krenz B. Deuschle K. Deigner T. Unseld S. Kepp G. Wege C.. (2015). Early function of the Abutilon mosaic virus AC2 gene as a replication brake. J. Virol. 89, 3683–3699. 10.1128/JVI.03491-14 25589661 PMC4403429

[B56] Kumar J. Kumar J. Singh S. P. Tuli R. (2014). Association of satellites with a mastrevirus in natural infection: complexity of wheat dwarf India virus disease. J. Virol. 88, 7093–7104. 10.1128/JVI.02911-13 24719407 PMC4054350

[B57] Kumar P. P. Usha R. Zrachya A. Levy Y. Spanov H. Gafni Y. (2006). Protein-protein interactions and nuclear trafficking of coat protein and βC1 protein associated with Bhendi yellow vein mosaic disease. Virus Res. 122, 127–136. 10.1016/j.virusres.2006.07.007 16934356

[B58] Kumar V. Mishra S. K. Rahman J. Taneja J. Sundaresan G. Mishra N. S.. (2015). Mungbean yellow mosaic Indian virus encoded AC2 protein suppresses RNA silencing by inhibiting Arabidopsis RDR6 and AGO1 activities. Virology 486, 158–172. 10.1016/j.virol.2015.08.015 26433748

[B59] Lozano G. Trenado H. P. Fiallo-Olivé E. Chirinos D. Geraud-Pouey F. Briddon R. W.. (2016). Characterization of non-coding DNA satellites associated with sweepoviruses (genus *Begomovirus, Geminiviridae*) - definition of a distinct class of begomovirus-associated satellites. Front. Microbiol. 7:162. 10.3389/fmicb.2016.00162 26925037 PMC4756297

[B60] Mansoor S. Khan S. H. Bashir A. Saeed M. Zafar Y. Malik K. A.. (1999). Identification of a novel circular single-stranded DNA associated with cotton leaf curl disease in Pakistan. Virology 259, 190–199. 10.1006/viro.1999.9766 10364503

[B61] Matić S. Pegoraro M. Noris E. (2016). The C2 protein of tomato yellow leaf curl Sardinia virus acts as a pathogenicity determinant and a 16-amino acid domain is responsible for inducing a hypersensitive response in plants. Virus Res. 215, 12–19. 10.1016/j.virusres.2016.01.014 26826600

[B62] Melgarejo T. A. Kon T. Rojas M. R. Paz-Carrasco L. Zerbini F. M. Gilbertson R. L. (2013). Characterization of a New World monopartite begomovirus causing leaf curl disease of tomato in ecuador and peru reveals a new direction in geminivirus evolution. J. Virol. 87, 5397–5413. 10.1128/JVI.00234-13 23468482 PMC3648196

[B63] Mizutani T. Daryono B. S. Ikegami M. Natsuaki K. T. (2011). First report of tomato leaf curl New Delhi virus infecting cucumber in Central Java, Indonesia. Plant Dis. 95:1485 10.1094/PDIS-03-11-0196 30731770

[B64] Mnari-Hattab M. Zammouri S. Belkadhi M. S. Doña D. B. ben Nahia E. Hajlaoui M. R. (2015). First report of tomato leaf curl New Delhi virus infecting cucurbits in Tunisia. New Dis. Rep. 31:21 10.5197/j.2044-0588.2015.031.021

[B65] Mubin M. Amin I. Amrao L. Briddon R. W. Mansoor S. (2010). The hypersensitive response induced by the V2 protein of a monopartite begomovirus is countered by the C2 protein. Mol. Plant Pathol. 11, 245–254 10.1111/j.1364-3703.2009.00601.x 20447273 PMC6640282

[B66] Nagendran K. Satya V. K. Mohankumar S. Karthikeyan G. (2016). Molecular characterization of a distinct bipartite begomovirus species infecting ivy gourd (*Coccinia grandis* L.) in Tamil Nadu, India. Virus Genes 52, 146–152. 10.1007/s11262-015-1278-6 26739457

[B67] Nawaz-ul-Rehman M. S. Mansoor S. Briddon R. W. Fauquet C. M. (2009). Maintenance of an old world betasatellite by a new world helper begomovirus and possible rapid adaptation of the betasatellite. J. Virol. 83, 9347–9355. 10.1128/JVI.00795-09 19570867 PMC2738271

[B68] Nawaz-ul-Rehman M. S. Nahid N. Mansoor S. Briddon R. W. Fauquet C. M. (2010). Post-transcriptional gene silencing suppressor activity of the two non-pathogenic alphasatellites associated with begomoviruses. Virology 405, 300–308. 10.1016/j.virol.2010.06.024 20598726

[B69] Noueiry A. O. Lucas W. J. Gilbertson R. L. (1994). Two proteins of a plant DNA virus coordinate nuclear and plasmodesmal transport. Cell 76, 925–932. 10.1016/0092-8674(94)90366-2 8124726

[B70] Padidam M. Beachy R. N. Fauquet C. M. (1995). Tomato leaf curl geminivirus from India has a bipartite genome and coat protein is not essential for infectivity. J. Gen. Virol. 76, 25–35. 10.1099/0022-1317-76-1-25 7844539

[B71] Padidam M. Beachy R. N. Fauquet C. M. (1996). The role of AV2 (“precoat”) and coat protein in viral replication and movement in tomato leaf curl geminivirus. Virology 224, 390–404. 10.1006/viro.1996.0546 8874500

[B72] Palanichelvam K. Kunik T. Citovsky V. Gafni Y. (1998). The capsid protein of tomato yellow leaf curl virus binds cooperatively to single-stranded DNA. J. Gen. Virol. 79, 2829–2833. 10.1099/0022-1317-79-11-2829 9820160

[B73] Panno S. Iacono G. Davino M. Marchione S. Zappardo V. Bella P. (2016). First report of tomato leaf curl New Delhi virus affecting zucchini squash in an important horticultural area of southern Italy. New Dis. Rep. 33:6 10.5197/j.2044-0588.2016.033.006

[B74] Pooma W. Petty I. T. D. (1996). Tomato golden mosaic virus open reading frame AL4 is genetically distinct from its C4 analogue in monopartite geminiviruses. J. Gen. Virol. 77, 1947–1951. 10.1099/0022-1317-77-8-1947 8760447

[B75] Qazi J. Amin I. Mansoor S. Iqbal J. Briddon R. W. (2007). Contribution of the satellite encoded gene βC1 to cotton leaf curl disease symptoms. Virus Res. 128, 135–139. 10.1016/j.virusres.2007.04.002 17482706

[B76] R Development Core Team (2016). R: A Language and Environment for Statistical Computing. Vienna: R Foundation for Statistical Computing Available online at: http://www.R-project.org/

[B77] Rodríguez-Negrete E. Lozano-Durán R. Piedra-Aguilera A. Cruzado L. Bejarano E. R. Castillo A. G. (2013). Geminivirus rep protein interferes with the plant DNA methylation machinery and suppresses transcriptional gene silencing. New Phytol. 199, 464–475. 10.1111/nph.12286 23614786

[B78] Rojas M. R. Hagen C. Lucas W. J. Gilbertson R. L. (2005). Exploiting chinks in the plant's armor: evolution and emergence of geminiviruses. Ann. Rev. Phytopathol. 43, 361–394. 10.1146/annurev.phyto.43.040204.135939 16078889

[B79] Rojas M. R. Jiang H. Salati R. Xoconostle-Cázares B. Sudarshana M. R. Lucas W. J.. (2001). Functional analysis of proteins involved in movement of the monopartite begomovirus, tomato yellow leaf curl virus. Virology 291, 110–125. 10.1006/viro.2001.1194 11878881

[B80] Rojas M. R. Noueiry A. M. Lucas W. J. Gilbertson R. L. (1998). Bean dwarf mosaic geminivirus movement proteins recognize DNA in a form- and size-specific manner. Cell 95, 105–113. 10.1016/S0092-8674(00)81786-9 9778251

[B81] Rosas-Díaz T. Macho A. P. Beuzón C. R. Lozano-Durán R. Bejarano E. R. (2016). The C2 protein from the geminivirus tomato yellow leaf curl sardinia virus decreases sensitivity to jasmonates and suppresses jasmonate-mediated defences. Plants 5:8. 10.3390/plants5010008 27135228 PMC4844413

[B82] Rothenstein D. Krenz B. Selchow O. Jeske H. (2007). Tissue and cell tropism of Indian cassava mosaic virus (ICMV) and its AV2 (precoat) gene product. Virology 359, 137–145. 10.1016/j.virol.2006.09.014 17049959

[B83] Rouhibakhsh A. Haq Q. M. I. Malathi V. G. (2011). Mutagenesis in ORF AV2 affects viral replication in Mungbean yellow mosaic India virus. J. Biosci. 36, 329–340. 10.1007/s12038-011-9041-1 21654086

[B84] Saeed M. (2008). Limitations observed in the use of agroinoculation for geminivirus research. Virus Genes 37, 434–435. 10.1007/s11262-008-0279-0 18770016

[B85] Saeed M. (2010). Tomato leaf curl New Delhi virus DNA a component and cotton leaf curl multan betasatellite can cause mild transient symptoms in cotton. Acta Virol. 54, 317–318. 10.4149/av_2010_04_317 21175259

[B86] Saeed M. Behjatnia S. A. A. Mansoor S. Zafar Y. Hasnain S. Rezaian M. A. (2005). A single complementary-sense transcript of a geminiviral DNA β satellite is determinant of pathogenicity. Mol. Plant-Microbe Interact. 18, 7–14. 10.1094/MPMI-18-0007 15672813

[B87] Saeed M. Mansoor S. Rezaian M. A. Briddon R. W. Randles J. W. (2008). Satellite DNA β overrides the pathogenicity phenotype of the C4 gene of tomato leaf curl virus, but does not compensate for loss of function of the coat protein and V2 genes. Arch. Virol. 153, 1367–1372. 10.1007/s00705-008-0124-8 18521533

[B88] Saeed M. Y. Zafar Y. Randles J. W. Rezaian M. A. (2007). A monopartite begomovirus-associated DNA β satellite substitutes for the DNA B of a bipartite begomovirus to permit systemic infection. J. Gen. Virol. 88, 2881–2889. 10.1099/vir.0.83049-0 17872543

[B89] Sambrook J. Frisch E. F. Maniatis T. (1989). Molecular Cloning: A Laboratory Manual. New York, NY: Cold Spring Harbor Laboratory Press.

[B90] Sánchez-Campos S. Martínez-Ayala A. Márquez-Martín B. Aragón-Caballero L. Navas-Castillo J. Moriones E. (2013). Fulfilling Koch's postulates confirms the monopartite nature of tomato leaf deformation virus: a begomovirus native to the New World. Virus Res. 173, 286–293. 10.1016/j.virusres.2013.02.002 23415858

[B91] Saunders K. Bedford I. D. Briddon R. W. Markham P. G. Wong S. M. Stanley J. (2000). A unique virus complex causes ageratum yellow vein disease. Proc. Natl. Acad. Sci. U.S.A. 97, 6890–6895. 10.1073/pnas.97.12.6890 10841581 PMC18771

[B92] Saunders K. Briddon R. W. Stanley J. (2008). Replication promiscuity of DNA-β satellites associated with monopartite begomoviruses; deletion mutagenesis of the ageratum yellow vein virus DNA-β satellite localises sequences involved in replication. J. Gen. Virol. 89, 3165–3172. 10.1099/vir.0.2008/003848-0 19008407

[B93] Saunders K. Norman A. Gucciardo S. Stanley J. (2004). The DNA β satellite component associated with ageratum yellow vein disease encodes an essential pathogenicity protein (βC1). Virology 324, 37–47. 10.1016/j.virol.2004.03.018 15183051

[B94] Saunders K. Salim N. Mali V. R. Malathi V. G. Briddon R. W. Markham P. G.. (2002). Characterisation of Sri Lankan cassava mosaic virus and Indian cassava mosaic virus: evidence for acquisition of a DNA B component by a monopartite begomovirus. Virology 293, 63–74. 10.1006/viro.2001.1251 11853400

[B95] Settlage S. B. See R. G. Hanley-Bowdoin L. (2005). Geminivirus C3 protein: replication enhancement and protein interactions. J. Virol. 79, 9885–9895. 10.1128/JVI.79.15.9885-9895.2005 16014949 PMC1181577

[B96] Shen W. Dallas M. B. Goshe M. B. Hanley-Bowdoin L. (2014). SnRK1 phosphorylation of AL2 delays cabbage leaf curl virus infection in *Arabidopsis*. J. Virol. 88, 10598–10612. 10.1128/JVI.00761-14 24990996 PMC4178870

[B97] Srivastava A. Kumar S. Jaidi M. Raj S. Shukla S. (2016). First report of tomato leaf curl New Delhi virus on opium poppy (*Papaver somniferum)* in India. Plant Dis. 100, 232 10.1094/PDIS-08-15-0883-PDN

[B98] Stanley J. Gay M. G. (1983). Nucleotide sequence of cassava latent virus DNA. Nature 301, 260–262. 10.1038/301260a0

[B99] Sunter G. Bisaro D. M. (1991). Transactivation in a geminivirus: AL2 gene product is needed for coat protein expression. Virology 180, 416–419. 10.1016/0042-6822(91)90049-H 1984661

[B100] Sunter G. Bisaro D. M. (1997). Regulation of a geminivirus coat protein promoter by AL2 protein (TrAP): evidence for activation and derepression mechanisms. Virology 232, 269–280. 10.1006/viro.1997.8549 9191840

[B101] Sunter G. Sunter J. L. Bisaro D. M. (2001). Plants expressing tomato golden mosaic virus AL2 or beet curly top virus L2 transgenes show enhanced susceptibility to infection by DNA and RNA viruses. Virology 285, 59–70. 10.1006/viro.2001.0950 11414806

[B102] Tahir M. Haider M. S. (2005). First report of tomato leaf curl New Delhi virus infecting bitter gourd in Pakistan. Plant Pathol. 54:807 10.1111/j.1365-3059.2005.01215.x

[B103] Trinks D. Rajeswaran R. Shivaprasad P. V. Akbergenov R. Oakeley E. J. Veluthambi K.. (2005). Suppression of RNA silencing by a geminivirus nuclear protein, AC2, correlates with transactivation of host genes. J. Virol. 79, 2517–2527. 10.1128/JVI.79.4.2517-2527.2005 15681452 PMC546592

[B104] Van Wezel R. Liu H. Tien P. Stanley J. Hong Y. (2001). Gene C2 of the monopartite geminivirus tomato yellow leaf curl virus-China encodes a pathogenicity determinant that is localized in the nucleus. Mol. Plant-Microbe Interact. 14, 1125–1128. 10.1094/MPMI.2001.14.9.1125 11551077

[B105] van Wezel R. Liu H. Wu Z. Stanley J. Hong Y. (2003). Contribution of the zinc finger to zinc and DNA binding by a suppressor of posttranscriptional gene silencing. J. Virol. 77, 696–700. 10.1128/JVI.77.1.696-700.2003 12477872 PMC140617

[B106] Vanitharani R. Chellappan P. Pita J. S. Fauquet C. M. (2004). Differential roles of AC2 and AC4 of cassava geminiviruses in mediating synergism and suppression of posttranscriptional gene silencing. J. Virol. 78, 9487–9498. 10.1128/JVI.78.17.9487-9498.2004 15308741 PMC506916

[B107] Wang H. Hao L. Shung C.-Y. Sunter G. Bisaro D. M. (2003). Adenosine kinase is inactivated by geminivirus AL2 and L2 proteins. Plant Cell 15, 3020–3032. 10.1105/tpc.015180 14615595 PMC282852

[B108] Yang X. Baliji S. Buchmann R. C. Wang H. Lindbo J. A. Sunter G.. (2007). Functional modulation of the geminivirus AL2 transcription factor and silencing suppressor by self-interaction. J. Virol. 81, 11972–11981. 10.1128/JVI.00617-07 17715241 PMC2168806

[B109] Zaidi S. S. A. Shafiq M. Amin I. Scheffler B. E. Scheffler J. A. Briddon R. W.. (2016). Frequent occurrence of tomato leaf curl New Delhi virus in cotton leaf curl disease affected cotton in Pakistan. PLoS ONE 11:e0155520. 10.1371/journal.pone.0155520 27213535 PMC4877078

[B110] Zerbini F. M. Briddon R. W. Idris A. Martin D. P. Moriones E. Navas-Castillo J.. (2017). ICTV virus taxonomy profile: geminiviridae. J. Gen. Virol. 98, 131–133. 10.1099/jgv.0.000738 28284245 PMC5802298

[B111] Zhang T. Luan J. B. Qi J. F. Huang C. J. Li M. Zhou X. P.. (2012). Begomovirus–whitefly mutualism is achieved through repression of plant defences by a virus pathogenicity factor. Mol. Ecol. 21, 1294–1304. 10.1111/j.1365-294X.2012.05457.x 22269032

[B112] Zhou X. (2013). Advances in understanding begomovirus satellites. Ann. Rev. Phytopathol. 51, 357–381. 10.1146/annurev-phyto-082712-102234 23915133

