# Supplementary material for: Maintenance of Cotton Leaf Curl Multan Betasatellite by Tomato Leaf Curl New Delhi Virus—Analysis by Mutation
Source: Front Plant Sci. 2017 Dec 22;8:2208. doi: 10.3389/fpls.2017.02208 (PMC5744040; doi:10.3389/fpls.2017.02208)
Supplement: Supplementary file 1 [file DataSheet1.DOCX]

Supplementary Material

**Maintenance of Cotton leaf curl Multan betasatellite by *Tomato leaf curl New Delhi virus -* analysis by mutation**

**Zafar Iqbal^1#*^, Muhammad Shafiq^1,2#^ Irfan Ali^1#^, Shahid Mansoor^1^, Rob W. Briddon^1^**

* **Correspondence**: [zafariqbal2009@gmail.com](mailto:zafariqbal2009@gmail.com) (ZI)

**Supplementary Figure 1.** Southern blot detection of CLCuMuB in *N. benthamiana* plants. The DNA extracts run on the gels were extracted from the leaves of plants inoculated with (A) TA and Cβ (1), TA, TB and Cβ (3-4), TA^ΔAV2^ and Cβ (5-7), TA^ΔAV2^, TB and Cβ (8-10); (B) TA and Cβ (1), TA, TB and Cβ (3-4), TA^ΔCP^ and Cβ (5-7), TA^ΔCP^, TB and Cβ (8-10); (C) TA and Cβ (2), TA, TB and Cβ (3-4), TA^ΔAC2^ and Cβ (5-7), TA^ΔAC2^, TB and Cβ (8-10),; (D) TA and Cβ (1), TA, TB and Cβ (3-4), TA^ΔAC4^ and Cβ (5-7), TA^ΔAC4^, TB and Cβ (8-10). The samples loaded in lane 1 of panel C and lanes 2 of panels A, B and D, consisted of 100ng of CLCuMuB plasmid (acc. no. AJ298903) as hybridization controls. The DNA forms of the plasmid are indicated as super-coiled (sc), and open-circular (oc). The sample loaded in lane H of panel C was extracted from a non-symptomatic (healthy) *N. benthamiana* plant. Samples from plants were extracted at 30 dpi and approximately equal amount of DNA (10µg) were loaded in each lane. For each blot a photograph of the ethidium bromide-stained genomic DNA bands on the agarose gel are shown below the blot to confirm equal loading.


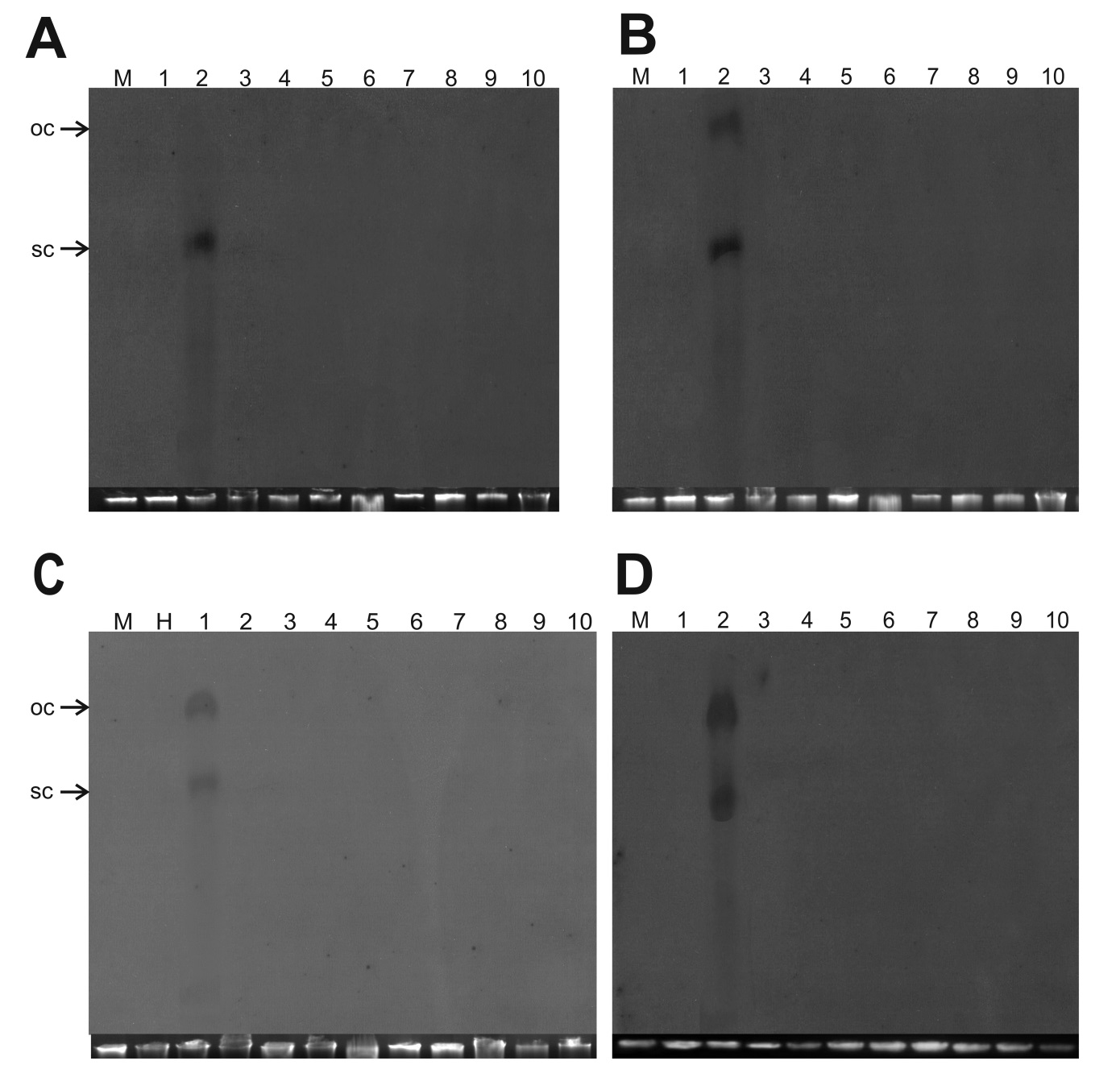


**Supplementary Table 1.** Quantification of ToLCNDV DNA A, DNA B and CLCuMuB in inoculated *N. benthamiana* plants by quantitative real-time PCR.

| **Inoculum*** | **TA (ng/µg of genomic DNA)** | **TB (ng/µg of genomic DNA)** | **Cβ (ng/µg of genomic DNA)** |
| --- | --- | --- | --- |
| *Gossypium arboreum* | 0.000 | 0.0000 | 0.000 |
| TA | 1.786 ±0.374 | ---- | ---- |
| TA+TB | 3.730 ±0.379 | 0.0003 ±0.00002 | ---- |
| TA+TB+Cβ | 5.040 ±0.162 | 0.0004 ±0.00002 | 1.920 ±0.134 |
| TA+ Cβ | 2.226 ±0.157 | ---- | 2.210 ±0.165 |
| TA^ΔCP^ | 1.003 ±0.132 | ---- | ---- |
| TA^ΔCP^+TB | 2.717 ±0.151 | 0.0001 ±0.00001 | ---- |
| TA^ΔCP^+TB+Cβ | 3.157 ±0.170 | 0.0001 ±0.00002 | 1.288 ±0.125 |
| TA^ΔCP^+Cβ | 2.150 ±0.164 | ---- | 1.326 ±0.151 |
| TA^ΔAV2^ | 1.653 ±0.186 | ---- | ---- |
| TA^ΔAV2^+TB | 4.236 ±0.241 | 0.0001 ±0.00002 | ---- |
| TA^ΔAV2^+TB+Cβ | 6.337 ±0.231 | 0.0003 ±0.00002 | 1.130 ±0.112 |
| TA^ΔAV2^+Cβ | 2.235 ±.329 | ---- | 1.250 ±0.150 |
| TA^ΔAC2^ | 1.945 ±0.189 | ---- | ---- |
| TA^ΔAC2^+TB | 3.527 ±0.099 | 0.0001 ±0.00001 | ---- |
| TA^ΔAC2^+TB+Cβ | 4.183 ±0.101 | 0.0001 ±0.00002 | 0.061 ±0.024 |
| TA^ΔAC2^+Cβ | 1.954 ±0.170 | ---- | 0.001 ±0.006 |
| TA^ΔAC4^ | 0.456 ±0.098 | ---- | ---- |
| TA^ΔAC4^+TB | 3.147 ±0.040 | 0.0001 ±0.00002 | ---- |
| TA^ΔAC4^+TB+Cβ | 2.425 ±0.022 | 0.0003 ±0.00002 | 1.842 ±0.122 |
| TA^ΔAC4^+Cβ | 1.530 ±0.164 | ---- | 2.312 ±0.181 |

The experiment was conducted in three technical repeats and the results shown are the means with standard deviation.

* Viruses, mutants and betasatellite are denoted as ToLCNDV DNA A (TA), ToLCNDV DNA B (TB), ToLCNDV DNA A bearing a mutation of the CP gene (TA^ΔCP^), ToLCNDV DNA A bearing a mutation of the AV2 gene (TA^ΔAV2^), ToLCNDV DNA A bearing a mutation of the TrAP gene (TA^ΔAC2^), ToLCNDV DNA A bearing a mutation of the AC4 gene (TA^ΔAC4^) and CLCuMuB (Cβ).

**Supplementary Table 2.** Statistical analysis to test the equality of proportions of infectivity of ToLCNDV mutants in *N. benthamiana* plants.

| **Inoculum** | **TA** | **Significance*** | **Cβ**  **Total infected plants/ Total inoculated plants** | **Significance*** |
| --- | --- | --- | --- | --- |
|  | **Total infected plants/ Total inoculated plants** |  |  |  |
| TA | 6/35 | B | - - | - - |
| TA,TB | 18/18 | A |  |  |
| TA^ΔAV2^ | 1/15 | A | - - | - - |
| TA^ΔCP^ | 1/15 | A | - - | - - |
| TA^ΔAC2^ | 8/15 | A | - - | - - |
| TA^ΔAC4^ | 7/11 | A | - - | - - |
|  |  |  |  |  |
| TA, Cβ | 15/24 | A | 9/24 | A |
| TA | 6/35 | B | - - | - - |
| TA^ΔAV2^, Cβ | 4/15 | B | 3/15 | A |
| TA^ΔCP^, Cβ | 12/14 | A | 8/14 | A |
| TA^ΔAC2^, Cβ | 8/15 | A | 0/15 | B |
| TA^ΔAC4^, Cβ | 9/13 | A | 0/13 | B |
|  |  |  |  |  |
| TA^ΔAV2^ | 1/15 | A | - - | - - |
| TA^ΔAV2^, Cβ | 4/15 | A | - - | - - |
|  |  |  |  |  |
| TA^ΔCP^ | 1/15 | B | - - | - - |
| TA^ΔCP^, Cβ | 12/14 | A | - - | - - |
|  |  |  |  |  |
| TA^ΔAC2^ | 8/15 | A | - - | - - |
| TA^ΔAC2^, Cβ | 8/15 | A | - - | - - |
|  |  |  |  |  |
| TA^ΔAC4^ | 7/11 | A | - - | - - |
| TA^ΔAC4^, Cβ | 9/13 | A | - - | - - |
|  |  |  |  |  |
| TA, Cβ | 7/9 | A | - - | - - |
| TA^ΔAV2^, Cβ | 4/15 | B | - - | - - |
| TA^ΔCP^, Cβ | 12/14 | A | - - | - - |
| TA^ΔAC2^, Cβ | 8/15 | A | - - | - - |
| TA^ΔAC4^, Cβ | 9/13 | A | - - | - - |
|  |  |  |  |  |
| TA, TB | 18/18 | A | - - | - - |
| TA^ΔAV2^, TB | 15/15 | A | - - | - - |
| TA^ΔCP^, TB | 14/14 | A | - - | - - |
| TA^ΔC2^, TB | 13/13 | A | - - | - - |
| TA^ΔC4^, TB | 9/9 | A | - - | - - |
|  |  |  |  |  |
| TA^ΔV2^, TB | 15/15 | A | - - | - - |
| TA^ΔV2^, TB, Cβ | 14/14 | A | - - | - - |
|  |  |  |  |  |
| TA^ΔCP^, TB | 14/15 | A | - - | - - |
| TA^ΔCP^, TB, Cβ | 14/15 | A | - - | - - |
|  |  |  |  |  |
| TA^ΔC2^, TB | 13/13 | A | - - | - - |
| TA^ΔC2^, TB, Cβ | 15/15 | A | - - | - - |
|  |  |  |  |  |
| TA^ΔC4^, TB | 9/9 | A | - - | - - |
| TA^ΔC4^, TB, Cβ | 15/15 | A | - - | - - |
|  |  |  |  |  |
| TA, TB and Cβ | 20/20 | A | 10/20 | A |
| TA^ΔV2^, TB, Cβ | 14/15 | A | 1/15 | B |
| TA^ΔCP^, TB, Cβ | 14/15 | A | 5/15 | A |
| TA^ΔC2^, TB, Cβ | 15/15 | A | 0/15 | B |
| TA^ΔC4^, TB, Cβ | 15/15 | A | 1/15 | B |

*****Values having the same letter are statistically not significantly different. Within each group the value(s) in grey are compared to the value in yellow (the control in each case). Values across groups are not compared.
